# Beyond Fuel: Exercise-Induced Lactate as a Metabolic-Epigenetic Regulator in Central Nervous System Health and Disease

**DOI:** 10.3390/biom16010043

**Published:** 2025-12-26

**Authors:** Boyi Zong, Fengzhi Yu, Fanghui Li, Peng Sun, Lin Li

**Affiliations:** 1 School of Sport Sciences, Nanjing Normal University, Nanjing 210046, China; 12211@njnu.edu.cn; 2 School of Exercise and Health, Shanghai University of Sport, Shanghai 200438, China; 2311516003@sus.edu.cn; 3 College of Physical Education and Health, East China Normal University, Shanghai 200241, China; lli@tyxx.ecnu.edu.cn; 4 Key Laboratory of Adolescent Health Assessment and Exercise Intervention of Ministry of Education, East China Normal University, Shanghai 200241, China

**Keywords:** exercise, central nervous system, lactate metabolism, lactate shuttle, G protein-coupled receptor 81, lactylation modification, neuroprotection

## Abstract

Lactate, as a pivotal metabolite generated by the body, has attracted considerable attention in numerous biological disciplines in recent years. In addition to its role in supplying energy, lactate also functions as a signaling molecule, with the capacity to mediate a diverse array of physiological effects. Within the central nervous system, lactate is involved in the regulation of critical physiological processes, including neurogenesis, synaptic plasticity, mitochondrial biogenesis, neuroinflammation, and cerebral angiogenesis. Furthermore, lactate has been implicated in the pathogenesis of several central nervous system diseases, such as Alzheimer’s disease, stroke, and spinal cord injury, among others. Physical exercise is recognized as a significant neuroprotective strategy; however, further research is required to elucidate the underlying biological mechanisms. In essence, the role of lactate as a metabolic-epigenetic core is gradually becoming a subject of increasing academic interest. The regulatory function of lactate is thought to involve its production (via lactate dehydrogenase), shuttle (via monocarboxylate transporters), sensing (via G protein-coupled receptor 81), and lactylation modifications, among others. This review synthesizes current evidence to elucidate the multifaceted roles of lactate in central nervous system physiology and pathology under exercise regulation, with a view to bridging the gap between molecular mechanisms and therapeutic potential, thereby paving the way for novel strategies in central nervous system disease intervention.

## 1. Introduction

Lactate is produced as a by-product of glucose metabolism through glycolysis [1]. It is well-documented that conditions such as high-intensity exercise and hypoxia inhibit the tricarboxylic acid (TCA) cycle, thus resulting in increased glycolytic flux and subsequent pyruvate conversion to lactate by lactate dehydrogenase (LDH) [2]. Within the central nervous system (CNS), it primarily originates from glycolysis in astrocytes, as well as from peripheral muscle tissue and other tissues [3]. Beyond its metabolic functions, lactate has been shown to act as a regulatory signaling molecule within the CNS. The substance exerts its influence on a variety of cell types, including neurons, astrocytes, neural stem/progenitor cells (NSCs/NPCs), microglia, oligodendrocytes (OLs), and endothelial cells (ECs) [4,5,6,7,8]. This regulatory mechanism exerts a substantial influence on pivotal physiological processes, including neurogenesis, synaptic plasticity, axonal regeneration, and angiogenesis, among others [9,10,11,12,13,14,15]. Furthermore, lactate is increasingly recognized as a significant biomarker for the diagnosis of CNS diseases [16,17,18]. Lactate and its associated pathways have been identified as promising innovative therapeutic targets for CNS diseases.

**Figure 1:**
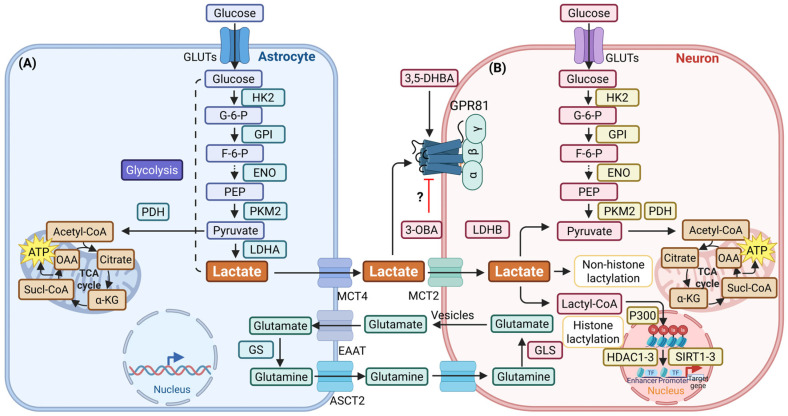
Schematic illustration of the astrocyte-neuron lactate shuttle. (**A**) Glucose undergoes glycolysis in astrocytes to produce lactate, which is then transported to neurons via MCTs. (**B**) In this location, it fulfils the roles of an energy substrate and a biological substrate for energy metabolism and lactylation. Furthermore, it functions as a signaling molecule, activating GPR81-related signaling pathways [20,41,42]. This figure was created using BioRender (https://app.biorender.com/, accessed on 24 November 2025). Abbreviations: ↑, increase or activation; ⊥, decrease or inhibition; ⇢, a brief presentation of the facilitation mechanism; ?, the effect has not been confirmed. 3,5-DHBA, 3,5-dihydroxybenzoic acid; 3-OBA, 3-hydroxybutyrate acid; α-KG, α-Ketoglutarate; Acetyl-CoA, Acetyl Coenzyme A; ATP, adenosine triphosphate; ASCT2, alanine-serine-cysteine transporter 2; EAAT, excitatory amino acid transporter; ENO, enolase; F-6-P, fructose-6-phosphate; G-6-P, glucose-6-phosphate; GLS, glutaminase; GLUT, glucose transporter; GPI, glucose phosphate isomerase; GPR81, G protein-coupled receptor 81; GS, glutamine synthetase; HDAC, histone deacetylase; HK2, hexokinase 2; LDH, lactate dehydrogenase; MCT, monocarboxylate transporters; NADH, nicotinamide adenine dinucleotide; OAA, oxaloacetic acid; PAX, pannexin; PEP, phosphoenolpyruvate; PFK, phosphofructokinase; PKM2, pyruvate kinase isozymes M2; SIRT, silent mating type information regulation 2 homolog; Sucl-CoA, Succinyl-CoA; TCA, tricarboxylic acid.

Monocarboxylate transporters (MCTs) are vital for the transportation of lactate between cells. High-affinity MCTs facilitate the uptake of lactate when intracellular concentrations are low. Conversely, low-affinity MCTs mediate its efflux to prevent excessive accumulation [19]. Within the CNS, the astrocyte-neuron lactate shuttle (ANLS) model serves to underscore the significance of lactate in metabolic coupling. MCT1 and MCT4 have been demonstrated to facilitate the export of lactate from astrocytes, while MCT2 has been shown to enable its uptake into neurons, where it serves as an oxidative energy substrate. Glutamate release during neuronal activity concurrently stimulates glucose uptake and glycolysis in astrocytes, promoting lactate production and sustaining this energy transfer cycle. This cycle is critical for supporting neuronal function during periods of high activity [20] (Figure 1). Moreover, the molecular mechanisms underlying the effects of lactate involve G protein-coupled receptor 81 (GPR81) signaling and lactylation modifications. Lactate has been demonstrated to function as an endogenous agonist for GPR81, predominantly binding to Gαi protein to inhibit adenylate cyclase (AC)/cyclic adenosine monophosphate (cAMP)/protein kinase A (PKA) signaling. Concurrently, it activates G protein-independent pathways such as β-arrestin signaling [21,22]. The *Gpr81* gene promoter region contains a conserved peroxisome proliferator-activated receptor (PPAR) response element, which facilitates the binding of PPARγ/retinoid X receptor heterodimers, thereby modulating transcription [23]. Synthetic agonists such as 3,5-dihydroxybenzoic acid (3,5-DHBA) have been identified [24]. However, the functional role of compounds such as 3-hydroxybutyrate (3-OBA), which have been proposed as antagonists or modulators, requires further validation [25,26]. Lactylation, a recently identified post-translational modification, involves the transfer of a lactyl group to lysine residues by an enzyme, thereby altering the function of the protein [27]. It has been demonstrated that the process of histone lactylation has the capacity to modify the structure of chromatin, transcription factor binding, and promoter accessibility. This, in turn, has the effect of regulating gene expression. Non-histone protein lactylation exerts its influence on protein function by means of altered interactions, enzymatic activity and subcellular localization [28,29]. This dynamic process is subject to regulation by various enzymes, including Writers such as P300, lysine acetyltransferase 2A (KAT2A), histone acetyltransferase binding protein 1, and KAT5; Readers, such as the Brahma-related gene-1 protein; and Erasers, such as histone deacetylase 1–3 (HDAC1–3), HDAC8, and silent mating type information regulation 2 homolog 1–3 (SIRT1–3) [30]. The functions of Writers, Readers and Erasers are pivotal in the catalysis of lactylation, the recognition of modifying groups, the regulation of the corresponding biological processes and the removal of modifying groups.

Physical exercise, as a safe, non-invasive intervention strategy with systemic benefits, has been extensively demonstrated through empirical research to exert broad and positive effects on the CNS. Its significant application potential in the prevention, delay, and adjunctive treatment of various CNS diseases, has been demonstrated [31,32]. Among the physiological changes induced by exercise, the dynamic regulation of lactate metabolism is of particular significance. It has been demonstrated that during periods of moderate-to-high intensity physical exercise, an accelerated process of glycolysis within skeletal muscle tissue leads to a substantial increase in the production of lactate. This phenomenon precipitates a swift escalation in blood lactate concentrations, which can attain levels that are multiple or even dozens of times the baseline level observed during periods of rest [33,34]. Furthermore, exercise systemically optimizes lactate shuttle and utilization efficiency across different tissues and organs (e.g., skeletal muscle, brain, heart, and liver) by upregulating the expression and activity of MCTs [35]. Recent breakthroughs have further elucidated the molecular basis of lactate signaling. Research findings demonstrate the ability of physical exercise to modulate the lactate-GPR81 axis. Activation of the lactate-GPR81 axis downstream modulates crucial signaling pathways, including AC/cAMP/PKA, thereby exerting precise regulatory effects on cellular energy homeostasis, inflammatory responses, and even cell survival [36,37,38]. Advances in epigenetics research have resulted in a growing understanding of the biological significance of lactylation modifications, including histone and non-histone lactylation, in the context of exercise physiology [39,40]. In consideration of the aforementioned advances, the present integrative review sought to synthesize current knowledge on the role of lactate in the physiology and pathology of the CNS, with a particular focus on the effects of exercise. In order to ensure comprehensive coverage of the field, a broad and iterative literature search was conducted using the PubMed and Web of Science databases. The focus of the search was on articles published up to October 2025. The search terms encompassed key concepts such as “lactate” or “lactic acid” combined with “central nervous system” or “brain” or “spinal cord”, “exercise” or “physical exercise” or “physical activity”. The reference lists of retrieved articles were also meticulously scanned for the purpose of identifying any additional relevant publications. The selection of literature was guided by the objective of constructing a coherent narrative on the topic, prioritizing seminal studies, recent breakthroughs, and papers contributing to mechanistic understanding across disciplines.

## 2. Exercise-Induced Lactate Modulation of Central Nervous System Physiology

**Figure 2:**
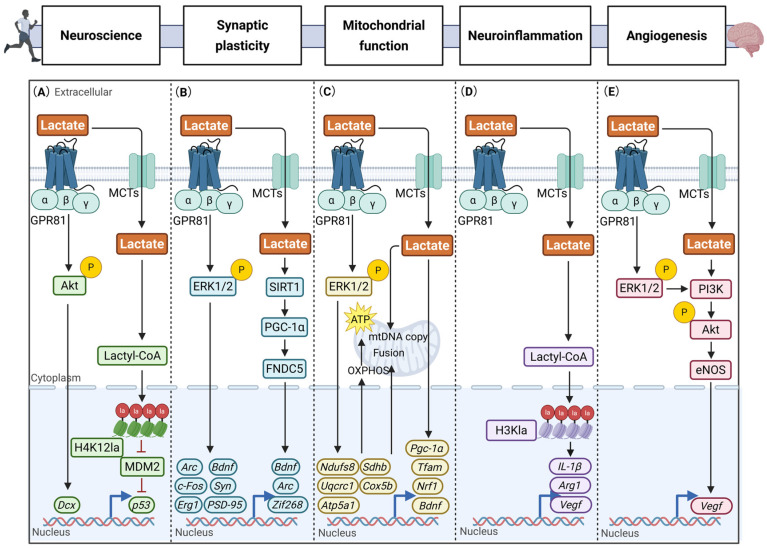
Neuroprotective effects of exercise-induced lactate and related mechanisms. (**A**) The activation of the lactate/GPR81 pathway by physical exercise has been demonstrated to promote neurogenesis [43]. This process may be related to the Akt/PKB signaling pathway. Furthermore, the process of exercise-induced lactate shuttling has been demonstrated to regulate histone lactylation, thereby influencing the proliferation of NSCs through the MDM2-p53 signaling pathway [9]. (**B**) Physical exercise has been observed to activate the lactate/GPR81/ERK1/2 pathway, thereby enhancing synaptic plasticity [44]. In addition, lactate has been observed to induce the activation of the SIRT1/PGC-1α/FNDC5/BDNF pathway, which further contributes to improved synaptic plasticity [45]. (**C**) The lactate/GPR81/ERK1/2 signaling pathway, which is activated by exercise, has been demonstrated to increase the number of hippocampal mitochondria. This phenomenon has been demonstrated to support oxidative phosphorylation, ATP production, mitochondrial biogenesis, and fusion [44]. Furthermore, the activation of PGC-1α by exercise has been linked to lactate signaling [46]. (**D**) Physical exercise has been shown to induce H3 histone lactylation in microglia, thereby suppressing their pro-inflammatory polarization [47]. (**E**) Physical exercise has been observed to stimulate lactate/GPR81/ERK1/2-PI3K/Akt signaling in brain outer membrane fibroblasts, thereby promoting cerebral angiogenesis [48]. Additionally, it has been demonstrated that physical exercise enhances angiogenesis through the activation of pathways such as Akt/eNOS/VEGF, which are critical for vascular development and repair [49]. This figure was created using BioRender (https://app.biorender.com/, accessed on 24 November 2025). Abbreviations: ↑, increase or activation; ⊥, decrease or inhibition; Akt, protein kinase B; Arc, activity-regulated cytoskeleton-associated protein; ARG1, Arginase-1; ATP, adenosine triphosphate; ATP5A1, ATP synthase, H^+^ transporting, mitochondrial F1 complex, α subunit 1; BDNF, brain-derived neurotrophic factor; c-Fos, c-fos proto-oncogene; COX5B, cytochrome c oxidase subunit 5B; DCX, doublecortin; NOS, endothelial nitric oxide synthase; ERK, extracellular regulated protein kinases; FNDC5, fibronectin type III domain-containing protein 5; GPR81, G protein-coupled receptor 81; IL, interleukin; MCT, monocarboxylate transporters; NDUFS8, NADH dehydrogenase (ubiquinone) Fe-S protein 8; NRF1, nuclear respiratory factor 1; NSC, neural stem cell; PGC-1α, peroxisome proliferator-activated receptor γ coactivator 1α; PI3K, phosphatidylinositol 3-kinase; PSD-95, postsynaptic density protein 95; SDHP, succinate dehydrogenase protein; SIRT, silent mating type information regulation 2 homolog; SYN, synaptophysin; TFAM, transcription factor A, mitochondrial; UQCRC1, ubiquinol-cytochrome c reductase core protein 1; VEGF, vascular endothelial growth factor.

Within the CNS, lactate fulfils a variety of functions in different cells, regulating a number of physiological processes. During periods of exercise, it plays a crucial role in mediating the exercise-induced promotion of neurogenesis, enhancement of synaptic plasticity, improvement of mitochondrial function, reduction in neuroinflammation, and increase in cerebral angiogenesis (Figure 2). The precise mechanisms by which exercise-induced lactate exerts its effects involve the actions of multiple signaling pathways. These include, but are not limited to, extracellular regulated protein kinases 1/2 (ERK1/2), SIRT1/peroxisome proliferators-activated receptor γ coactivator lα (PGC-1α)/fibronectin type III domain-containing protein 5 (FNDC5), and protein kinase B (PKB, i.e., Akt)/endothelial nitric oxide synthase (eNOS)/vascular endothelial growth factor (VEGF), among others (Figure 2).

### 2.1. Exercise-Induced Lactate Modulation of Neurogenesis

Neurogenesis, the process by which functional neurons are generated from NSCs, occurs constitutively within specific neurogenic niches of the mammalian brain, most notably the subventricular zone (SVZ) of the lateral ventricles and the subgranular zone (SGZ) of the hippocampal dentate gyrus (DG), persisting into adulthood. The process comprises several stages, including stem cell activation and proliferation, neuronal commitment and fate specification, migration, neuronal differentiation and maturation, and functional integration [50,51]. The regulation of classical neurogenesis is governed through a highly orchestrated interplay of niche-derived signals, including essential growth factors, conserved developmental morphogenic pathways, activity-dependent neurotransmitters, and intrinsic transcriptional programs [52]. Extensive research has confirmed that regular exercise is a crucial intervention for promoting adult hippocampal neurogenesis (AHN) [53,54]. It has been demonstrated that exercise exerts a substantial impact on the proliferation of NPCs, the survival and differentiation of newly generated neurons, and the integration of their functional circuits. The structural basis for this brain-enhancing effect is closely linked to multiple molecular and cellular adaptations that are induced by exercise. These include upregulation of pivotal neurotrophic factors, such as brain-derived neurotrophic factor (BDNF), optimized regulation of neurotransmitter and hormonal systems, enhanced local cerebral blood flow (CBF) and angiogenesis, and systemic anti-inflammatory and antioxidant effects [55]. A number of factors are secreted into the bloodstream during exercise, including lactate, and these may act as key mediators of exercise-induced neurogenesis. During the process of neurogenesis, MCT1 and MCT2 have been observed to regulate lactate efflux and influx, respectively, within mouse NSCs. This regulatory process is pivotal in maintaining intracellular homeostasis [9]. It was determined that a decrease in L-lactate levels in mice with a knockout of pyruvate kinase M2 (PKM2) led to impaired AHN and cognitive function. In contrast, exogenous L-lactate supplementation has been shown to restore AHN and cognitive function via MCT2-dependent mechanisms [10]. Research has demonstrated that following its entry into cells via MCT2, lactate activates the promotion of NPC proliferation through the phosphorylation of ERK1/2 and Akt signaling pathways [56]. However, the loss or diminished expression of the *Mct2* gene has been demonstrated to impair hippocampal neurogenesis and long-term memory in animal models [57,58] (Table 1). Furthermore, studies have shown that the suppression of *Mct2* gene expression can reverse the neurogenesis- and cognitive function-promoting effects of voluntary wheel running and treadmill exercise on the hippocampus of rodents [9,59]. It is evident that glycolytic enzyme-mediated lactate production and MCT-mediated lactate transport play crucial roles in exercise-induced neurogenesis. Notably, other studies have found that selective knockout of *Mct1* gene in ECs leads to lactate accumulation in the hippocampal region and impairs neurogenesis [5]. These findings suggest that lactate may have a dual role in the process of neurogenesis, depending on the concentration range. Within this range, a moderate increase in lactate levels has been shown to promote neurogenesis, whereas excessive accumulation may exert opposite effects.

The role and mechanisms of lactate in neurogenesis via GPR81-mediated signaling pathways are also noteworthy. Research has indicated that physical training or exogenous L-lactate supplementation can promote neurogenesis by activating GPR81 in rodent brains [43,60]. Further research revealed that the effects of HIIT on neurogenesis in the V-SVZ region are contingent on GPR81 activation, whereas its influence on neurogenesis in the hippocampal SGZ region is independent of GPR81 activation [61]. Thus, GPR81 signaling may exert region-specific effects on neurogenesis, warranting further investigation into the underlying mechanisms for the differential HIIT responses between the V-SVZ and hippocampus. Furthermore, research has indicated that lactate has the capacity to influence neuronal differentiation independently of the GPR81 signaling pathway [62]. As demonstrated by Hwang et al. [63], the supplementation of exogenous lactate in combination with moderate-intensity treadmill running led to improvements in reference memory, retention memory, and spatial working memory in mice. The observed effect was likely to have been mediated by a combination of factors, including enhanced activation of the hippocampal PGC-1α/FNDC5/BDNF pathway and upregulation of MCT2 expression. However, the combined intervention has not resulted in a significant alteration in the levels of GPR81 protein expression in the hippocampus. The findings, which appear to be in opposition to one another, indicate that GPR81’s function in lactate-mediated neurogenesis may be subject to high context dependency, which may involve unidentified post-receptor compensatory signaling mechanisms or crucial pathways that are independent of GPR81 (Figure 2A). Furthermore, lactate functions as a substrate for H4K12la, modulating the quiescence of NSCs via the mouse double minute 2 homolog (MDM2)-P53 pathway [9], and promoting microtubule dynamics via HDAC6-mediated α-tubulin lactylation to support neuronal maturation [64]. Despite the confirmation of the involvement of lactylation modifications in the regulation of neurogenesis, the function of exercise within this process remains to be elucidated. It is imperative that future research employs comprehensive bioinformatics analysis techniques to systematically elucidate the complexity of exercise and lactate regulation of neurogenesis and their integrative mechanisms.

### 2.2. Exercise-Induced Lactate Modulation of Synaptic Plasticity

Neurons have been observed to demonstrate a clear preference for the use of lactate as an energy substrate during periods of activity, often prioritizing its utilization over glucose. Intensification of neural activity (e.g., high-frequency discharge) results in a rapid increase in local brain lactate levels. This, in turn, provides neurons with immediate ATP to support energy-intensive physiological processes such as ion pump operation, neurotransmitter release, and reuptake [65,66]. Furthermore, lactate has been demonstrated to provide essential energy support during synapse plasticity-related events, including structural remodeling (i.e., synaptic protein synthesis and dendritic spine remodeling) [11,67,68] (Table 2). It is vital to note that the presence of lactate, which is derived from astrocytes, and is transported via MCT4, is found to be of critical importance in ensuring that synaptic integrity remains consistent within neurons. The suppression of its expression has been demonstrated to result in a reduction in dendritic spine density within the motor cortex, a decline in synaptic protein expression, and a diminution in neuronal activity [69]. Following its transport into neurons via MCT2, lactate is converted into pyruvate by LDH1 within the cytoplasm. This process entails the reduction in nicotinamide adenine dinucleotide (NAD^+^) to nicotinamide adenine dinucleotide (reduced form) (NADH). The impact of NADH on N-methyl-d-aspartate (NMDA) receptor function is determined by its effect on the cellular redox state. Elevated levels of NADH have been demonstrated to facilitate the maintenance of the reduced state of key sulphhydryl groups on the receptor protein, thereby supporting or enhancing its baseline function. This enhancement has been shown to trigger an influx of Ca^2+^, which in turn activates several signaling pathways, including those involving ERK1/2, mitogen-activated protein kinase (MAPK), calcium/calmodulin-dependent protein kinase II (CaMKII), and PKA. Consequently, this results in the expression of synaptic plasticity-related genes, including activity-regulated cytoskeletal (Arc), c-Fos, and early growth response protein 1 (EGR1) [70,71]. Mechanistically, lactate has also been shown to promote exercise-induced BDNF expression via the SIRT1/PGC-1α/FNDC5 pathway, thereby enhancing synaptic transmission and cognitive function [45]. It is noteworthy that the inhibition of MCTs results in the reversal of exercise-induced neuroprotective benefits, thereby underscoring the pivotal function of lactate shuttle in regulating synaptic plasticity. In mouse models demonstrating insulin resistance, a significant decrease in the expression of the *Mct1* and *Mct4* genes has been observed within the hippocampus, accompanied by a decline in MCT2 expression. This is accompanied by elevated blood lactate levels and ANLS dysfunction, reduced expression of synaptic proteins such as postsynaptic density 95 (PSD-95) and synaptophysin (SYN), and decreased Akt/mammalian target of rapamycin (mTOR) phosphorylation levels, suggesting synaptic dysfunction. The implementation of progressive resistance exercise has been demonstrated to engender a restoration of blood lactate balance, an upregulation of MCT expression, and a re-establishment of mTOR, NMDAR, and SYN protein levels. Consequently, this has been shown to improve hippocampal synaptic plasticity [72]. In addition, it has been demonstrated that regular moderate-intensity treadmill exercise exerts a positive effect on the enhancement of hippocampal long-term potentiation (LTP) and an increase in dendritic complexity in aged mice. Furthermore, it has been demonstrated to upregulate MCT4, BDNF, glutamine synthetase, and full-length TrκB expression, thereby enhancing learning and memory functions [73].

Beyond its role in energy metabolism, lactate also modulates presynaptic neurotransmitter release and neuronal excitability by activating GPR81 [74,75]. This mechanism involves crosstalk between GPR81 and other GPCRs, including γ-aminobutyric acid type B receptor (GABA_B_R), adenosine A1, and α2A-adrenergic receptors [74]. In the CA3 region of the hippocampus, lactate or its analog 3,5-DHBA has been observed to enhance synaptic plasticity via a signaling cascade involving Gβγ, PI3K, PKC and CaMKII [76]. Observations of an HIIT program have indicated that there is an upregulation of the expression of BDNF, EGR1, and PSD-95 in the hippocampus. Concurrently, there is activation of ERK1/2 in a GPR81-dependent manner. However, this effect is completely abolished in *Gpr81* gene knockout mice [44] (Figure 2B). In summary, lactate exerts a synergistic effect on the modulation of synaptic plasticity through MCT-mediated transport and GPR81-regulated signaling pathways (Table 2). It is recommended that future studies concentrate on elucidating its specific mechanisms across different brain regions, cell types, physiological and pathological contexts. Furthermore, priority should be given to the precise correlation between exercise intervention parameters (frequency, intensity, and duration) and lactate dynamics.

**Table 2:** The effects and mechanisms of lactate on synaptic plasticity within the central nervous system.

Subjects and Samples	Treatments	Mechanism	Major Effects of Lactate	Reference
Amnesia rat hippocampus tissues	*Mct1*, *Mct2*, and *Mct4* gene manipulation; L-lactate or D-lactate administration	MCT-dependent manner	Phosphorylation of CREB, Arc, and cofilin ↑; LTP ↑; long-term memory formation ↑	[77]
Amnesia rat hippocampus tissues	*Mct1*, *Mct2*, and *Mct4* gene manipulation; L-lactate administration	MCT-dependent manner	Arc/Arg3.1 expression ↑; long-term memory formation ↑	[78]
Diabetic mouse hippocampus tissues	L-lactate administration	MCT-dependent manner	Arc, ERG1, and BDNF expression ↑	[79]
Mouse hippocampus tissues	*Gpr81* gene manipulation; L-lactate or 3Cl-HBA administration	Activation of GPR81	Neuronal excitability ↓; EPSP ↓; PPP ↑	[65]
Epileptic human, mouse and rat brain tissues; primary granule cells and cortical neurons	*Gpr81* gene manipulation; 3Cl-HBA administration	Activation of GPR81	Spontaneous neuronal Ca^2+^ spiking and EPSP ↓	[80]
TBI rat cortex and hippocampus tissues	L-lactate administration	Activation of GPR81 and MCT2	PSD-95, GAP43, BDNF, and TrκB expression ↑	[81]
Rat hippocampus tissue	L-lactate administration	Activation of GPR81, PI3K, PKC and CaMKII signaling	Synapse-specific potentiation ↑; PPP ↑; coefficient of variation ↓; EPSP-to-spike coupling ↑	[76]
Mice; primary cortical neurons	*Gpr81* gene manipulation; L-lactate, 3,5-DHBA, and 3Cl-HBA administration	Activation of GPR81 and AC/cAMP/PKA signaling	Spontaneous neuronal Ca^2+^ spiking, firing frequency, and EPSP ↓; PPP ↑	[74]
Mouse primary astrocytes	*β-arrestin2* gene manipulation; L-lactate administration	Activation of GPR81-β-arrestin2-MAPK signaling	Arc/Arg3.1 expression ↑	[22]
Mouse primary cortical neurons and glial cells; HEK293T cells	*CB1* gene manipulation; L-lactate or 3,5-DHBA administration	Activation of GPR81 and NMDAR	Synaptic d-serine availability ↑	[68]
Mouse primary cortical neurons	L-lactate administration	Activation of NMDAR and downstream ERK1/2 signaling	*Arc*, *c-Fos*, and *Zif268* gene expression ↑	[71]
Mouse primary cortical neurons	L-lactate administration	Activation of NMDAR	*Arc*, *Bdnf*, *Erg1/2/3/4*, *Fos*, *Npas4*, *Nr4a3*, *Rgs4*, *Adcyap1*, *Vegfa*, *Gfra2*, *Nr4a2* gene expression ↑; *Bcl2l11*, *Apaf1*, *Txnip*, and *Hrk* gene expression ↓	[70]
Mouse brainstem-spinal cord	L-lactate administration	Activation of calcium permeable channels, L-type Ca^2+^ channels and NMDAR	Glutamatergic synapses ↑; respiratory motor outflow ↑	[11]
POCD rat hippocampus tissues	L-lactate and/or EX-527 administration	Activation of SIRT1 signaling	BDNF, Arc, and Erg1 expression ↑	[82]
Rat primary cortical neurons	*Mct2* gene manipulation; L-lactate administration	Activation of Akt/GSK-3β pathway	Axon length, dendrite length and number ↑	[83]

Abbreviations: ↑, increase or activation; ↓, decrease or inhibition; AC, adenylate cyclase; Akt, protein kinase B; APAF1, apoptotic peptidase activating factor 1; Arc, activity-regulated cytoskeleton-associated protein; Arg3.1, activity-regulated gene 3.1; Adcyap1, pituitary adenylate cyclase-activating polypeptide 1; BDNF, brain-derived neurotrophic factor; Bcl2l11, BCL2 like 11; cAMP, cyclic adenosine monophosphate; CaMKII, calcium/calmodulin-dependent protein kinase II; CB1, cannabinoid receptor 1; CREB, cAMP response element-binding protein; EPSP, excitatory postsynaptic potential; ERG1, early growth response protein 1; ERK1/2, extracellular signal-regulated kinases 1 and 2; GAP43, growth-associated protein 43; GPR81, G protein-coupled receptor 81; Gfra2, GDNF family receptor α 2; GSK-3β, glycogen synthase kinase 3β; Hrk, harakiri, BCL2 interacting protein; LTP, long-term potentiation; MAPK, mitogen-activated protein kinase; MCT, monocarboxylate transporter; NMDAR, N-methyl-D-aspartate receptor; NPAS4, neuronal PAS domain protein 4; NR4A2, nuclear receptor subfamily 4 group A member 2; PI3K, phosphoinositide 3-kinase; PKA, protein kinase A; PKC, protein kinase C; PPP, paired-pulse potentiation; PSD-95, postsynaptic density protein 95; RGS4, regulator of G-protein signaling 4; TBI, traumatic brain injury; TrκB, tropomyosin receptor kinase B; TXNIP, thioredoxin interacting protein; VEGFA, vascular endothelial growth factor A; Zif268, zinc finger protein 268.

### 2.3. Exercise-Induced Lactate Modulation of Brain Mitochondrial Function

Within the CNS, mitochondria serve not only as cellular powerhouses but also play a pivotal role in maintaining neuronal function and survival by regulating critical processes such as calcium homeostasis, reactive oxygen species (ROS) balance, and apoptosis [84,85]. A substantial body of research demonstrated that physical exercise exerts a significant effect on mitochondrial function, with a variety of outcomes being observed. These include enhanced mitochondrial biogenesis, improved kinetic balance, strengthened autophagy quality control, and ultimately elevated oxidative phosphorylation (OXPHOS) efficiency and antioxidant capacity. This effect is partially mediated by lactate and its associated signaling pathways. Recent studies have indicated that lactate activates PGC-1α, thereby increasing mitochondrial DNA copy number and upregulating the expression of crucial OXPHOS components, such as the ATP synthase β subunit (ATPB) and cytochrome c. This, in turn, has been shown to enhance bioenergetic output [79,86,87]. Furthermore, lactate has been demonstrated to promote mitochondrial fusion by increasing the expression of mitochondrial fusion proteins 1/2 (MFN1/2) and decreasing the expression of dynamin-related protein 1 (DRP1) and fission protein 1 (FIS1). This process serves to enhance the efficiency of ATP synthesis. Furthermore, lactate has also been demonstrated to elevate the expression of regulatory factors, namely SIRT3 and oxidation resistance 1 (OXR1), thereby enhancing antioxidant defense capabilities [88,89] (Table 3). The results of the animal studies demonstrate that acute exercise or HIIT significantly elevates levels of lactate in the hippocampus, accompanied by upregulation of MCT1/2, PGC-1α, and BDNF expression. This, in turn, has been shown to promote mitochondrial biogenesis and OXPHOS function, thus improving mitochondrial kinetic balance [46,88]. The findings emphasize the pivotal role of lactate in regulating mitochondrial function (Table 3), and underscore its potential as a therapeutic target for exercise-induced improvements in mitochondrial health and neuroprotection.

It has been demonstrated that HIIT promotes lactate activation of GPR81, thereby enhancing mitochondrial biogenesis, fusion, and ATP production through the ERK1/2 signaling pathway. This effect is absent in *Gpr81* knockout mice, indicating that GPR81 is an essential component for exercise-regulated mitochondrial function [44] (Figure 2C). Furthermore, the extracellular pH decrease induced by lactate metabolism activates acid-sensitive ion channel 1a, triggering calcium signaling that enhances mitochondrial lactate breakdown and suppresses ROS production [90]. Fibroblast growth factor 21 (FGF-21) has also been demonstrated to participate in the regulation of the lactate-pyruvate metabolic axis. Its neuroprotective effects are closely linked to improved mitochondrial function and reduced lactate accumulation [91]. The pivotal role of the exercise-lactate regulatory axis in brain mitochondrial energy metabolism, kinetic balance, and quality control has now been preliminarily elucidated. Subsequent research should concentrate on deciphering the mitochondrial response specificity to lactate signaling across different brain regions and cell types, whilst also delving deeper into the regulatory functions of lactylation.

**Table 3:** The effects and mechanisms of lactate on brain mitochondrial function within the central nervous system.

Subjects and Samples	Treatments	Mechanism	Major Effects of Lactate	Reference
Diabetic mouse hippocampus tissues	L-lactate administration	MCT-dependent manner	ROS production ↓; SOD activity ↑; respiratory chain complex I activity ↑; ATP production ↑	[79]
Rat hippocampus tissues	4-CIN administration	MCT2-dependent manner	OXPHOS ↑	[92]
Rat ACC tissues	L-lactate administration	MCT2 and NMDAR-dependent manner	PGC-1α, SIRT3, and ATPB expression ↑; mitochondrial biogenesis ↑	[86]
Rat hippocampus tissues	L-lactate administration	Activation of SIRT3 signaling	PGC-1α, SIRT3, KIF5B, OXR1, PYGM, ATG7, CAMK2G, ATPB and Cyt-c expression ↑; mtDNA copy number ↑	[89]
Mouse hippocampal and cortical neuron; CHO cells	*Asic1a* and *Mct2* gene manipulation; L-lactate administration	Activation of ASIC1a	Cytosolic and mitochondrial Ca^2+^ signals in neurons ↑; mitochondrial respiration ↑; mitochondrial ROS production ↓	[90]
Mice; primary cortical neurons	*Aralar* gene manipulation; L-lactate administration	Activation of ARALAR	Cytosolic ATP/ADP ratio ↑; mitochondrial ROS production ↓	[93]
Mouse primary cortical neurons	L-Lactate, pyruvate, D-lactate, and D-Glucose administration	Activation of P2Y/KATP/PI3K cascade	Mitochondrial oxidative metabolism ↑; mitochondrial ATP formation ↑	[94]
Human glioblastoma cell lines and Zebrafish microglia	L-lactate administration	Crosstalk with IGFBP6	*Pgc-1α*, *Tfam*, *Cox IV*, *Cox II*, *Cytb*, and *Nd-4* gene expression ↑; mitochondrial biogenesis and OXPHOS ↑	[95]

Abbreviations: ↑, increase or activation; ↓, decrease or inhibition; ACC, anterior cingulate cortex; 4-CIN, α-cyano-4-hydroxycinnamic acid; ARALAR, mitochondrial aspartate-glutamate carrier; ASIC1a, acid-sensing ion channel 1a; ATP, adenosine triphosphate; ATPB, ATP synthase subunit β; ATG7, autophagy related 7; CAMK2G, calcium/calmodulin-dependent protein kinase II γ; CHO, Chinese hamster ovary; Cox II, cytochrome c oxidase subunit II; Cox IV, cytochrome c oxidase subunit IV; Cyt-c, cytochrome c; IGFBP6, insulin-like growth factor binding protein 6; KATP, ATP-sensitive potassium channel; KIF5B, kinesin family member 5B; mtDNA, mitochondrial DNA; Nd-4, NADH dehydrogenase subunit 4 (mitochondrially encoded); OXPHOS, oxidative phosphorylation; OXR1, oxidation resistance 1; P2Y, purinergic receptor P2Y; PGC-1α, peroxisome proliferator-activated receptor γ coactivator 1α; PYGM, glycogen phosphorylase, muscle associated; ROS, reactive oxygen species; SIRT3, silent mating type information regulation 2 homolog 3; SOD, superoxide dismutase; TFAM, mitochondrial transcription factor A; TGF-β, transforming growth factor β.

### 2.4. Exercise-Induced Lactate Modulation of Neuroinflammation

Neuroinflammation is defined as a highly complex immunopathological response within the CNS, characterized by the abnormal activation of microglia and astrocytes, the infiltration of peripheral immune cells, and the excessive release of multiple inflammatory mediators such as cytokines, chemokines, and ROS. The persistent disturbance of this process ultimately results in neuronal injury and cognitive decline through multiple mechanisms, including the induction of synaptic dysfunction, the disruption of blood-brain barrier (BBB) integrity, and the triggering of excitotoxicity [96]. The classical neuroinflammatory pathways are centered on the pattern recognition receptors of microglia. These molecules have the capacity to recognize pathogen or damage-associated molecular patterns, which in turn activates key signaling cascades such as nuclear factor κB (NF-κB) and MAPK. The process has been demonstrated to result in the release of pro-inflammatory cytokines, including tumor necrosis factor α (TNF-α), interleukin 1β (IL-1β), and IL-6, while concomitantly promoting NLR family pyrin domain protein 3 (NLRP3) inflammasome assembly. This pro-inflammatory state is subject to precise regulation by anti-inflammatory pathways, which are mediated by IL-10, TGF-β, and PPARγ. It has been demonstrated that these pathways guide microglial polarization towards a protective phenotype and facilitate inflammation resolution [97]. It is important to note that, as microglia age, there is an alteration in their metabolic characteristics. This includes changes in extracellular acidification rates, lactate concentrations and MCT expression [98]. A substantial body of research has indicated that lactate displays dual regulation in neuroinflammation, exhibiting both concentration-dependent and context-dependent properties. Under the physiological conditions, lactate has been demonstrated to elicit anti-inflammatory effects through a variety of mechanisms. For instance, lactate pretreatment suppresses LPS-induced glial cell protrusion retraction and, by activating the Akt signaling pathway, downregulates the expression of pro-inflammatory factors such as IL-6, TNF-α, and IL-1β while inhibiting NLRP3 inflammasome activation [99,100] (Table 4). However, it has been demonstrated that excessive lactate generation can intensify inflammatory responses, including the promotion of neuronal lipid deposition and the release of inflammatory cytokines [101]. At the epigenetic level, lactylation has been shown to emerge as a novel regulatory mechanism. For instance, lactate has been shown to stimulate H3K9la, thereby enhancing M1 phenotype polarization of microglia under inflammatory conditions [102]; conversely, lactylation of the p53 protein has been demonstrated to synergize with NF-κB signaling to amplify production of factors including iNOS, IL-6, IL-1β, and TNF-α in hypoxic microglia [103]. Moderate exercise has been demonstrated as an effective strategy for alleviating neuroinflammation [104,105]. The results of experimental research conducted on animals indicate that exogenous lactate supplementation in combination with regular treadmill training has a significant effect on the cognitive function. In addition, the supplementation has been shown to inhibit pro-inflammatory glial cell polarization and induce histone H3 lactylation. This, in turn, has been demonstrated to promote the transition of microglia towards the anti-inflammatory M2 phenotype. In vitro studies further corroborate the finding that exogenous lactate increases LPS-induced lactate levels in microglia and upregulates M2-type markers such as arginase 1 and VEGF expression [47] (Figure 2D). This finding suggests that exercise-induced lactate may influence the expression of microglial genes through lactylation, thereby modulating their immune functions.

Recent research has revealed the pivotal role of the lactate transporter and receptor in neuroinflammation. MCT1 has been demonstrated to upregulate 6-phosphofructo-2-kinase/fructose-2,6-bisphosphatase 3 (PFKFB3) expression via hypoxia-inducible factor 1α (HIF-1α), thereby accelerating gluconeogenesis in microglia and promoting their polarization towards the M1 phenotype. Conversely, the inhibition of MCT1 has been shown to reduce lactate transport, downregulate PFKFB3, and thereby mitigate LPS-induced expression of inducible nitric oxide synthase (iNOS), IL-1β, IL-6, and signal transducer and activator of transcription 1 (STAT1) phosphorylation [106]. In addition to microglia, astrocytes are also significant immune-related cells within the CNS. The administration of lactate has been demonstrated to facilitate the restoration of disrupted metabolic coupling between astrocytes and neurons. This process is accompanied by the inhibition of astrocyte activation via SIRT1, thereby contributing to the restoration of BBB integrity through the upregulation of endothelial MCT1 and GPR81 expression [107,108]. Recent research has indicated that chronic stress can instigate myeloid-biased hematopoiesis and proliferation of Ly6Chigh monocytes, thereby initiating inflammation of the hippocampus. This process, known as peripheral immune cell infiltration, has been demonstrated to occur as a result of such stress. Exercise has been shown to impede this process by activating the lactate-GPR81 axis, thereby ameliorating stress-related behavioral phenotypes [37]. These findings have illuminated the intricate and nuanced regulatory functions of lactate in the context of neuroinflammation, and revealed that lactate exerts significant concentration- and context-dependent effects through multifaceted mechanisms, encompassing metabolic regulation and epigenetic modification at various levels (Table 4). It is imperative that future research employs in-depth analysis at the cell-specific level of the dynamic response networks of lactate signaling across different neuroimmune cells. This will elucidate the relative contributions of receptor-dependent and -independent pathways under physiological and pathological conditions.

**Table 4:** The effects and mechanisms of lactate on neuroinflammation within the central nervous system.

Subjects and Samples	Treatments	Mechanism	Major Effects of Lactate	Reference
N9 microglial cell line and human microglia clone 3 cell line	*Gpr81* gene manipulation; L-lactate administration	Activation of GPR81	Microglial phagocytosis ↓; dysfunctional hyperactivated M1 state ↑; *Tnf-α*, *iNOS*, *Il-1β* and *Nlrp3* gene expression ↑	[109]
Acute seizures mice; HT22 cells	L-lactate, glutamate, and kainic acid administration	Activation of GPR81	Activation of microglia ↓; *Il-1β*, *Tnf-α* and *Il-6* gene expression ↓; apoptosis and neuronal injury ↓	[110]
BV-2 and SH-SY5Y cells	OGD and OGD/R; L-lactate and 3,5-DHBA administration	Activation of GPR81	OGD-induced intracellular acidification and TNF-α release ↓	[111]
Mouse primary cortical neuroglial cells	OGD/R; L-lactate administration	Inhibition of intracellular Ca^2+^ signaling	Apoptosis and necrosis ↓; *Il-1β*, *Tnf-α*, *Cox2*, *Cas-1*, *Mlkl*, *Nf-κB* gene expression ↓; *Il-10*, *Bcl-xL*, and *Ripk1* gene expression ↑	[112]
BV2, HEK293T cells, and primary microglia	LPS administration	Lactate-induced p53Kla and activation of NF-κB signaling	*Tnf-α*, *Il-6*, *IL1β*, and *iNOS* gene expression ↑; activation of BV2 cells ↑	[103]
Mouse HACE model; BV2 cells	*Ldha* gene manipulation; LPS administration	Lactate-induced NuRD complex lactylation and activation of NF-κB signaling	LPS/hypoxia-induced *Il-6*, *Il-1β* and *Tnf-α* gene expression ↑	[113]
POCD rat hippocampus tissues	L-lactate and EX-527 administration	Activation of SIRT1 signaling	Activation of microglia and astrocytes ↓; IL-1β and IL-6 expression ↓	[82]
MCAO mice; BV2 cells	L-lactate and YC-1 administration	Activation of HIF-1α and inhibition of NF-κB signaling	Number of CD86^+^ Iba1^+^ cells ↓; number of CD206^+^ Iba1^+^ cells ↑; iNOS expression ↓; Arg1 expression ↑; *iNOS*, *CD86*, *Il-6*, and *Tnf-α* gene expression ↓; *Tgf-β* and *Il-10* gene expression ↑; *Ccl-7* gene expression in BV2 cells	[114]
Mice; BV2 cells	OGD; *Hif-1*α gene manipulation; L-lactate and YC-1 administration	Activation of HIF-1α and inhibition of NF-κB signaling	iNOS expression ↓; Arg1 expression ↑; the ratio of *iNOS*/*CD206*, *CD86*/*Ym1*, *Il-6*/*Il-10* and *Tnf-α*/*Il-10* gene expression ↓	[115]
Mouse primary microglia	LPS and L-lactate administration	Phosphorylation of Akt	LPS-induced *Il-1β*, *Tnf-α*, and *Il-6* gene expression ↓; *Il-4*, *Il-10*, and *CD206* gene expression ↑; cell elongation ↑	[99]
Human glioblastoma cell lines and Zebrafish microglia	L-lactate administration	Crosstalk with IGFBP6	*Arg1*, *CD206*, *CD163*, *Tgf-β*, and *Il-6* gene expression ↑; microglia M2 polarization	[95]
Mouse primary microglia	LPS and L-lactate administration	N/A	LPS-induced IL-1β and TNF-α expression ↓; phosphorylation of NF-κB ↓; NLRP3 expression ↓	[100]
Trigeminal neuralgia mice	*Ldh* gene manipulation; L-lactate administration	N/A	*Il-1β*, *Tnf-α* and *Il-6* gene expression ↓	[116]
Rat primary astroglia and microglia	NH_4_Cl and L-lactate administration	N/A	Release of TNF-α, IL-6 and IL-1β ↑	[117]

Abbreviations: ↑, increase or activation; ↓, decrease or inhibition; Akt, protein kinase B; Arg1, arginase 1; Bcl-xL, B-cell lymphoma extra-large; Cas-1, caspase-1; Ccl-7, C-C motif chemokine ligand 7; COX2, cyclooxygenase-2; GPR81, G protein-coupled receptor 81; HACE, high-altitude cerebral edema; HEK293T, human embryonic kidney 293T cell line; HIF-1α, hypoxia-inducible factor 1α; iNOS, inducible nitric oxide synthase; Iba1, ionized calcium-binding adapter molecule 1; IGFBP6, insulin-like growth factor binding protein 6; IL, interleukin; Ldha, lactate dehydrogenase A; LPS, lipopolysaccharide; MCAO, middle cerebral artery occlusion; MLKL, mixed lineage kinase domain like pseudokinase; NF-κB, nuclear factor κB; NH4Cl, ammonium chloride; NLRP3, NLR family pyrin domain containing 3; NuRD, nucleosome remodeling deacetylase complex; OGD/R, oxygen-glucose deprivation and reoxygenation; p53Kla, p53 lysine lactylation; POCD, postoperative cognitive dysfunction; RIPK1, receptor-interacting serine/threonine-protein kinase 1; SIRT1, Silent mating type information regulation 2 homolog 1; TGF-β, transforming growth factor β; TNF-α, tumor necrosis factor α; YC-1, 3-(5′-hydroxymethyl-2′-furyl)-1-benzyl indazole.

### 2.5. Exercise-Induced Lactate Modulation of Cerebral Angiogenesis

Cerebral angiogenesis is defined as the physiological process of new blood vessel formation within the pre-existing vascular network of the brain, facilitating the supply of oxygen and nutrients to the tissue. The fundamental mechanism underpinning this process involves the multiple signaling pathway, including but not limited to VEGF/VFGFR2, angiopoietin (Ang)/Tie2, and Notch signaling. These pathways have been demonstrated to promote the proliferation of ECs, their migration, and the formation of luminal structures. Furthermore, pericytes and astrocytes provide structural support and nutritional signals to the neovasculature through direct contact and the secretion of paracrine factors such as TGF-β and platelet-derived growth factor B (PDGF-B), thereby ensuring the maintenance of vascular stability and BBB function [118,119]. Physiological angiogenesis is a fundamental basis for the cerebral adaptation to metabolic demands, the regulation of local blood flow, and the support of neurogenesis and cognitive function. The dysregulation of this process has been demonstrated to be closely associated with the onset and progression of various CNS diseases [119]. Recent studies have revealed the pivotal role of lactate in cerebral vascular development and dysfunction regulation. Systematic single-cell metabolic state analysis has revealed that proliferating radial glial progenitors generate substantial lactate via glycolysis during development [120] (Table 5). Further transcriptomic and spatial transcriptomic analyses revealed that peripheral sensory stimulation upregulates MCT2 expression in cortical neurons and MCT1 expression in ECs, accompanied by elevated intracerebral lactate levels, increased astrocytic VEGFA expression, and accelerated angiogenesis. A series of functional experiments confirmed that the neuronal MCT2 is indispensable in the mediation of stimulus-induced angiogenesis and metabolic responses. The mechanism may involve lactate influx, which is known to promote neuronal-astrocyte metabolic coupling. This, in turn, is believed to activate the astrocyte HIF-1α-VEGFA signaling axis [121]. These findings suggest that lactate metabolism and transport may exert a pro-angiogenic role during angiogenesis (Table 5).

It has been demonstrated that physical exercise promotes cerebral vascularization through mechanisms that are dependent on GPR81. The distribution of this receptor has been found to be concentrated within the cerebral microvasculature, particularly in medullary fibroblast-like and pericytes-like cells [122]. This action has been demonstrated to upregulate VEGFA expression, while suppressing the anti-angiogenic thrombospondin-1 (TSP-1). The knockout of the *Gpr81* gene has been demonstrated to result in microvascular developmental abnormalities, reduced pro-angiogenic factor expression, and elevated inflammatory markers [15]. The functionality of this pathway has been corroborated by exercise intervention studies. The validity of this mechanism is further supported by experimental evidence, namely that exogenous lactate supplementation in combination with HIIT has been shown to increase VEGFA expression and capillary density in mouse brains [122]. Moreover, this effect is reversible in *Gpr81* gene knockout models. In addition, it has been demonstrated that prolonged treadmill training activates GPR81-dependent ERK1/2-PI3K/Akt signaling in the brain, while HIIT and lactate intervention also activate the Akt/eNOS/VEGF pathway in the aged hippocampus, collectively promoting angiogenesis and improving cerebral blood perfusion [48,49] (Figure 2E). It is imperative to transcend the limitations of research that is predominantly focused on the GPR81-VEGF axis, establishing a comprehensive systemic network model of lactate regulation in vascular integrity. This will elucidate its functions in cellular regulation, basement membrane remodeling, and maintenance of hemodynamic homeostasis. Concurrently, exploration of the bidirectional regulatory mechanisms of lactate signaling in both pathological angiogenesis and physiological neovascularization will facilitate the development of context-dependent interventions targeting cerebral vascular remodeling.

**Table 5:** The effects and mechanisms of lactate on cerebral angiogenesis within the central nervous system.

Subjects and Samples	Treatments	Mechanism	Major Effects of Lactate	Reference
U251, SW1088, and HBMECs	*Mct1* and *Mct4* gene manipulation; L-lactate and AR-C155858 administration	MCT1-dependent manner	EC proliferation, migration and vessel assembly ↑; angiogenesis ↑	[123]
OIR Mice; HMC3 cells and HRMECs	*YY1* gene manipulation	Lactate-induced YY1K183la	FGF-2 expression in microglia ↑; EC proliferation and migration ↑; angiogenesis ↑	[124]
Mice; primary neurons	*Gpr81* gene manipulation; L-lactate administration	Activation of GPR81	TSP-1 expression ↓; VEGF, Ang-1, Ang-2, and PDGF expression ↑	[15]
Mice	*Gpr81* gene manipulation; L-lactate administration; HIIT	Activation of GPR81 and ERK1/2 and Akt signaling	VEGF expression ↑; capillary density ↑; angiogenesis ↑	[122]
Mice; radial glial progenitors	*Ldha* and *Ldhb* gene manipulation; L-lactate, GSK2837808A, and SR13800 administration	Activation of CXCL1 signaling	Vessel development ↑	[120]
ICH rats	L-lactate administration	Activation of NF-κB signaling	VEGF and bFGF expression ↑; angiogenesis ↑	[125]

Abbreviations: ↑, increase or activation; ↓, decrease or inhibition; Akt, protein kinase B; Ang-1, angiopoietin 1; bFGF, basic fibroblast growth factor; CXCL1, Chemokine (C-X-C Motif) ligand 1; EC, endothelial cell; ERK, extracellular signal-regulated kinase; FGF-2, fibroblast growth factor 2; GPR81, G protein-coupled receptor 81; HBMECs, human brain microvascular endothelial cells; HIIT, high-intensity interval training; HMC3, human microglial clone 3 cell line; HRMECs, human retinal microvascular endothelial cells; ICH, intracerebral hemorrhage; LDH, lactate dehydrogenase; MCT, monocarboxylate transporter; NF-κB, nuclear factor κB; OIR, oxygen induce retinopathy; PDGF, platelet-derived growth factor; TSP-1, thrombospondin-1; VEGF, vascular endothelial growth factor; YY1, Yin Yang 1.

## 3. The Role of Lactate in CNS Diseases and Exercise Intervention

The role of lactate in CNS diseases, and the underlying mechanisms by which it exerts its effects, have recently been the subject of increased research. The mechanisms through which exercise exerts its effects on CNS diseases, such as Alzheimer’s disease (AD), Ischemic stroke (IS), and spinal cord injury (SCI), as facilitated by lactate and associated pathways, encompass the modulation of brain energy metabolism, the elevation of neurotrophic support system levels, the enhancement of synaptic plasticity, and the reduction in neuroinflammation (Table 6). The multifaceted beneficial effects of this process are primarily attributed to changes in circulating and brain lactate levels, MCTs and GPR81 expression, and alterations in lactylation modifications.

### 3.1. Lactate in Alzheimer’s Disease and Exercise Intervention

**Figure 3:**
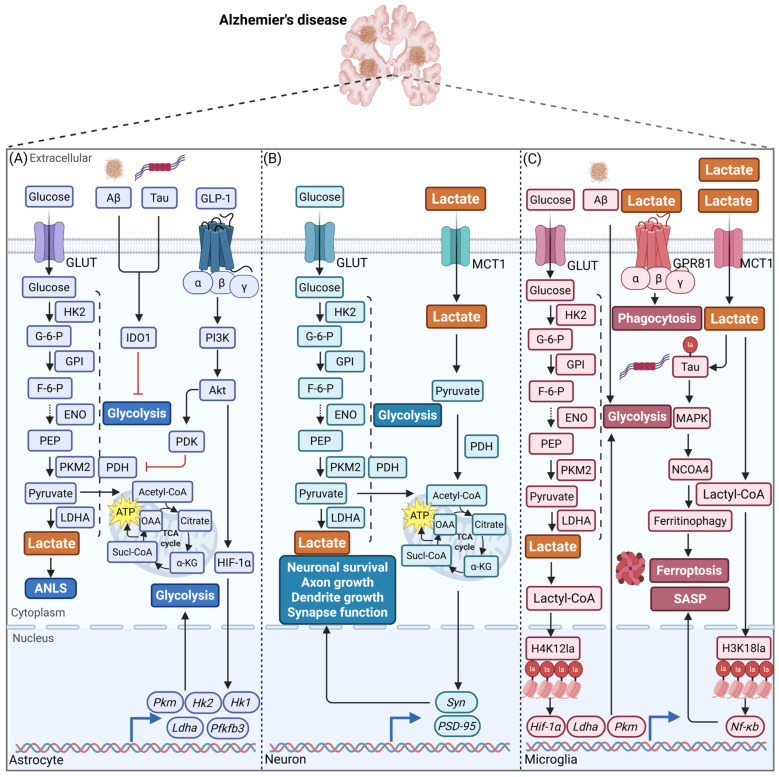
The regulatory role of lactate in the pathology of Alzheimer’s disease and the mechanisms involved. (**A**) In astrocytes, the presence of Aβ and tau proteins has been shown to activate the KYN pathway by increasing IDO1 expression. This activation has been shown to result in impaired astrocytic metabolism, disrupted lactate flux, and reduced neuronal support [139]. GLP-1 has been demonstrated to attenuate the Aβ-induced decline in astrocytic glycolysis through the activation of the PI3K/Akt pathway, leading to reduced OXPHOS levels, lower ROS production, and enhanced astrocytic support for neurons [158]. (**B**) In neurons, the GLP-1-induced astrocytic lactate release, which improved neuronal survival. This process has been shown to promote axon and dendrite growth and enhance synaptic function [158]. (**C**) In microglia, lactate has been shown to regulate phagocytosis through the activation of GPR81 [109]. Furthermore, H4K12la has been implicated in disease progression by regulating key glycolytic enzyme genes [162]. H3K18la has been observed to modulate inflammation-associated pathways, such as NF-κB [165]. The lactylation of the K677 site on tau proteins has been observed to influence ferritinophagy and ferroptosis via the MAPK pathway [168]. This figure was created using BioRender (https://app.biorender.com/, accessed on 24 November 2025). Abbreviations: ↑, increase or activation; ⊥, decrease or inhibition; ⇢, a brief presentation of the facilitation mechanism; Aβ, amyloid β-protein; Akt, protein kinase B; ATP, adenosine triphosphate; GLP-1, glucagon-like peptide 1; GLUT, glucose transporter; GPR81, G protein-coupled receptor 81; HIF-1α, hypoxia inducible factor 1α; IDO1, indoleamine-2,3-dioxygenase 1; KYN, kynurenine; LDHA, lactate dehydrogenase A; MCT, monocarboxylate transporters; MAPK, mitogen-activated protein kinase; NCOA4, nuclear receptor coactivator 4; NF-κB, nuclear factor κB; PDH, pyruvate dehydrogenase; PDK, pyruvate dehydrogenase kinase; PI3K, phosphatidylinositol 3-kinase; PKM2, pyruvate kinase M2; ROS, reactive oxygen species; SASP, senescence-associated secretory phenotype; TRP, tryptophan.

AD is the most prevalent cause of dementia. It is primarily characterized by the deposition of amyloid β-protein (Aβ), which forms age-related plaques, and the hyperphosphorylation of tau proteins, leading to neurofibrillary tangles (NFTs) [135]. Notably, the progression of AD is frequently accompanied by impaired glucose metabolism, resulting in abnormal central and peripheral lactate fluctuations [16,136,137,138,139]. Mechanistic studies have suggested that Aβ may trigger lactate release in the hippocampus [140], and promote astrocytic glycolysis and enhance lactate production by activating microglia, which in turn stimulates the Akt/mTOR/HIF-1α signaling pathway [141]. In accordance with this finding, research conducted on both human and animal subjects has demonstrated that, during specific phases of AD, lactate levels are elevated in particular regions of the brain and in cerebrospinal fluid (CSF) [142,143,144,145,146]. Concurrently, the expression of MCTs was also significantly elevated [147]. However, it should be noted that certain studies have also documented a decline in brain lactate levels and MCTs expression [148,149,150,151,152,153,154]. A reduction in intracerebral lactate levels and the expression of MCTs may result in impaired transport of lactate from glial cells to neurons, potentially resulting in neuronal lactate deficiency. Exogenous lactate supplementation has been demonstrated to restore lactate levels in the hippocampus and CSF, thereby enhancing synaptic plasticity and improving cognitive function (Figure 3B). Furthermore, the activity of enzymes such as β-secretase (BACE-1) and a disintegrin and metalloprotease 10 (ADAM10), which are implicated in Aβ processing, has been shown to be regulated as a consequence [127,155]. Additionally, lactate has been demonstrated to influence Aβ phagocytosis by microglia through the activation of the GPR81 [109] (Figure 3C). A substantial body of research has demonstrated that interventions which target discrete molecular pathways can effectively ameliorate AD-associated functional impairments by modulating lactate metabolism. For instance, the inhibition of indoleamine 2,3-dioxygenase 1 (IDO1) has been demonstrated to reverse the inhibition of astrocytic glycolysis induced by Aβ and tau, thereby enhancing hippocampal synaptic plasticity in an MCT-dependent manner [139]. Conversely, upregulating the vestigial-like family member 4 (VGLL4)-LDHA axis has been shown to promote lactate production, thereby alleviating amyloid precursor protein (APP) accumulation [156]. Furthermore, substances such as curcumin, FGF-21, and glucagon-like peptide-1 (GLP-1) enhance ANLS function by elevating lactate levels, regulating hepatic-cerebral lactate metabolism, or activating the PI3K/Akt pathway, respectively. This has been demonstrated to enhance energy supply and antioxidant capacity, thereby exerting neuroprotective effects [150,157,158] (Figure 3A). Collectively, these observations imply that lactate metabolism is intricately linked to multiple aspects of AD pathology, including energy deficits, inflammation, and Aβ dynamics.

Research indicates that lactylation modifications exert multi-level, multi-target regulatory effects in the pathological progression of AD, emerging as a promising intervention target [159,160]. At the epigenetic level, specific histone lactylation modifications (e.g., H4K12la and H3K18la) have been observed to activate glycolysis-related genes (e.g., *Pkm2*) or inflammatory pathways (e.g., NLRP3 and NF-κB). This process establishes a positive feedback loop that has been shown to promote Aβ deposition, tau hyperphosphorylation, and neuroinflammation. Research findings have demonstrated that the effective modulation of these lactylation levels can serve as a means of delaying pathological progression and enhancing cognitive function in affected individuals [161,162,163,164,165] (Figure 3C). With regard to non-histone modifications, reduced APP lysine-612 lactylation (APP-K612la) has been demonstrated to impede endosomal-lysosomal degradation, thus promoting Aβ generation. Conversely, enhancing lactylation at this site through mutation or lactate intervention has been demonstrated to promote APP endocytosis and reduce Aβ deposition [166]. It is noteworthy that tau protein lactylation exerts a dual effect, which has significant implications for the progression of tauopathy. Elevated lactylation at the K331 site has been found to enhance phosphorylation/cleavage and inhibit ubiquitination, thereby exacerbating tauopathy [167]; meanwhile, reduced lactylation at the K677 site has been shown to inhibit ferroptosis [168]. The findings reveal how lactylation modifies disease progression in AD by regulating multiple critical pathways, including Aβ metabolism, tau pathology, and neuroimmune responses. This finding suggests that early intervention targeting specific lactylation sites may offer novel strategies for the precision treatment of AD.

In recent years, an increasing body of research has confirmed the involvement of lactate in the bidirectional regulation of the brain-gut axis in AD. The levels and functions of the organism under consideration are profoundly influenced by a number of factors, including the dynamic effects of gut microbiota composition, dietary interventions, and disease progression. A significant increase in lactate-producing genera such as Bifidobacterium has been documented in patients diagnosed with AD, thereby indicating a structural dysbiosis of the gut microbiota. This increase has been demonstrated to be negatively associated with patients’ cognitive function scores [169]. However, it should be noted that this association exhibits context-dependent properties. Large-scale Mendelian randomization analyses further suggest, from a genetic perspective, a causal association between reduced systemic lactate levels and heightened AD risk [170], highlighting the potential significance of gut-derived lactate metabolic disruption in AD pathogenesis. In animal studies, following intervention with the modified Mediterranean-ketogenic diet (MkD), elevated fecal and serum lactate levels were found to correlate positively with enhanced intestinal barrier function and reduced hippocampal neuroinflammatory markers [171], suggesting that lactate may exert protective effects under specific interventions. At the mechanistic level, basic research provides molecular explanations for the bidirectional regulation of lactate. On the one hand, lactate has been shown to mediate beneficial signaling through GPR81. For instance, MkD-induced elevated lactate levels have been shown to enhance GPR81 expression in the brain, accompanied by increased levels of both lactate and neurotransmitter precursors (e.g., glutamine), suggesting potential neuroprotective effects via receptor signaling and energy metabolism regulation [171]. In contrast, within the pathological environment of AD, excessive colonization of specific pathogenic strains (e.g., Lactobacillus murinus overexpressing Rib adhesin) in the gut leads to elevated levels of lactate production. This, in turn, has been demonstrated to stimulate serum amyloid A production by activating the signaling pathway of the intestinal epithelial GPR81-NF-κB, thus driving peripheral Th1 immune inflammatory responses and exacerbating neuroinflammation [172]. In contrast, the pharmaceutical compound GV-971 has been shown to correct aberrant lactate production and its associated pathological processes by preventing the adhesion of the aforementioned strain in the intestinal tract. To conclude, lactate fulfils a dual function within the AD gut-brain axis. Under physiological or beneficial interventions, the moderate levels of lactate have been observed to exert anti-inflammatory and neuroprotective effects through pathways such as GPR81. Conversely, within the context of severe dysbiosis-induced lactate metabolism disruption, particularly excessive lactate production driven by specific pathogens, it accelerates disease progression through pro-inflammatory signaling. This bidirectional action underscores the critical importance of maintaining lactate metabolic homeostasis for preserving gut-brain axis homeostasis and intervening in AD.

It has been suggested that physical exercise exerts a positive regulatory effect on cognitive function, with lactate potentially serving as a pivotal mediating factor. The relevant human studies demonstrate that acute combined aerobic and resistance exercise has been shown to enhance cognitive flexibility. The enhancement in executive function is facilitated by blood lactate levels [173]. The effectiveness of HIIT in improving inhibitory control capacity is known to decrease with training adaptation, a phenomenon that has been associated with dynamic changes in cerebral lactate utilization [174]. Meta-regression analyses provide further corroboration of the concept that post-exercise lactate levels partially explain variance in inhibitory control [175]. Furthermore, acute exercise has been observed to elevate blood lactate levels in older adults and AD patients, concomitant with a reduction in cerebral grey matter glucose metabolism, accompanied by cognitive improvements [126,176]. In animal models, lactate has been demonstrated to be transported across the BBB into the brain, where it has been shown to promote learning and memory that is dependent on the hippocampus, in a BDNF-dependent manner [45]. The implementation of both HIIT and moderate-intensity continuous training has been demonstrated to alleviate symptoms of anxiety and depression, while concomitantly enhancing cognitive function in AD mice. This effect is accompanied by a remodeling of brain metabolic processes [177]. Mechanistically, the presence of lactate in the circulation following exercise is known to enter neurons via the MCT2. This process has been demonstrated to regulate the expression of genes associated with synaptic plasticity, including members of the Eph receptor family. It has been demonstrated that this results in the inhibition of abnormal synaptic phagocytosis by microglia through the GPR81/cAMP/PKA pathway, thereby protecting synaptic structure [128,178]. Furthermore, it has been demonstrated that exercise-induced lactate accumulation modulates BACE1 and ADAM10 activity, thereby shifting APP processing towards non-Aβ-generating pathways [127]. At the epigenetic level, treadmill exercise has been shown to induce H3 histone lactylation in microglia, thereby suppressing their pro-inflammatory polarization and consequently improving the neuroinflammatory microenvironment [47] (Table 6). Furthermore, the correlation between reduced hippocampal MCT2 expression in diabetic models and memory impairment has been demonstrated. Moreover, it has been shown that light and moderate intensity exercise can reverse this abnormality by restoring glycogen metabolism [179,180,181]. The combination of HIIT and semaglutide has been shown to enhance neuronal morphology and cognitive function in *db*/*db* mice. This mechanism involves lactate upregulating BDNF via the adenosine monophosphate-activated protein kinase (AMPK) and PKA-related pathway while reducing Aβ and p-tau levels [182]. The lactate-SIRT1-forkhead box protein O3 (FoxO3)-PTEN-induced kinase (PINK1)/Parkin pathway has also been demonstrated to participate in the clearance of Aβ and tau proteins, thereby delaying disease progression [183]. Current research has indicated the multi-pathway mediating role of lactate in the improvement of cognitive function induced by exercise in AD patients, encompassing energy substrate substitution, synaptic integrity maintenance, regulation of Aβ metabolism, and control of neuroinflammation. Subsequent research should further integrate cell-specific genetic manipulation techniques with multi-omics approaches to elucidate the dynamic functions of lactate signaling across disease stages and its interactive networks with other metabolites. Concurrently, it is imperative to distinguish the characteristic dynamic responses of lactate under varying exercise intensities and modalities.

### 3.2. Lactate in Stroke and Exercise Intervention

Stroke is defined as an acute, focal neurological impairment that cannot be attributed to any cause other than a cerebrovascular event [184]. The classification of strokes is typically into two primary categories: ischemic stroke (IS) and hemorrhagic stroke, with IS being the more prevalent form [185]. IS is characterized by the occlusion of cerebral arteries, resulting in reduced CBF and subsequent damage to neuronal and glial cells. It has been demonstrated that such disruption may result in secondary injuries, including the breakdown of the BBB and hemorrhagic transformation during reperfusion. In contrast, hemorrhagic stroke is distinguished by the rupture of an intracerebral artery, leading to cerebral hemorrhage [186]. Elevated cerebral lactate levels during the acute phase of stroke have been shown to reflect the severity of the ischemic event. Furthermore, such levels have been demonstrated to serve as a compensatory energy source. The persistence of elevated levels of lactate in the subacute phase has been shown to indicate the activation of aerobic glycolysis, a process which is required to meet the metabolic demands that arise subsequent to the initial injury [17,187,188,189,190]. In models of middle cerebral artery occlusion (MCAO), the exogenous administration of L-lactate has been shown to significantly reduce infarct volume and improve neurological functional outcomes, thereby demonstrating clear therapeutic potential. This effect is partially mediated through enhanced ANLS function and improved collateral blood flow perfusion [191,192,193,194]. MCTs have been demonstrated to play a pivotal role in mediating the aforementioned protective effects. For instance, electroacupuncture has been shown to promote lactate efflux for neuronal utilization by upregulating astrocytic MCT1 expression [195], whilst activating neuronal MCT2 has been demonstrated to alleviate cognitive deficits through AMPK-dependent mitochondrial biogenesis [196].

**Figure 4:**
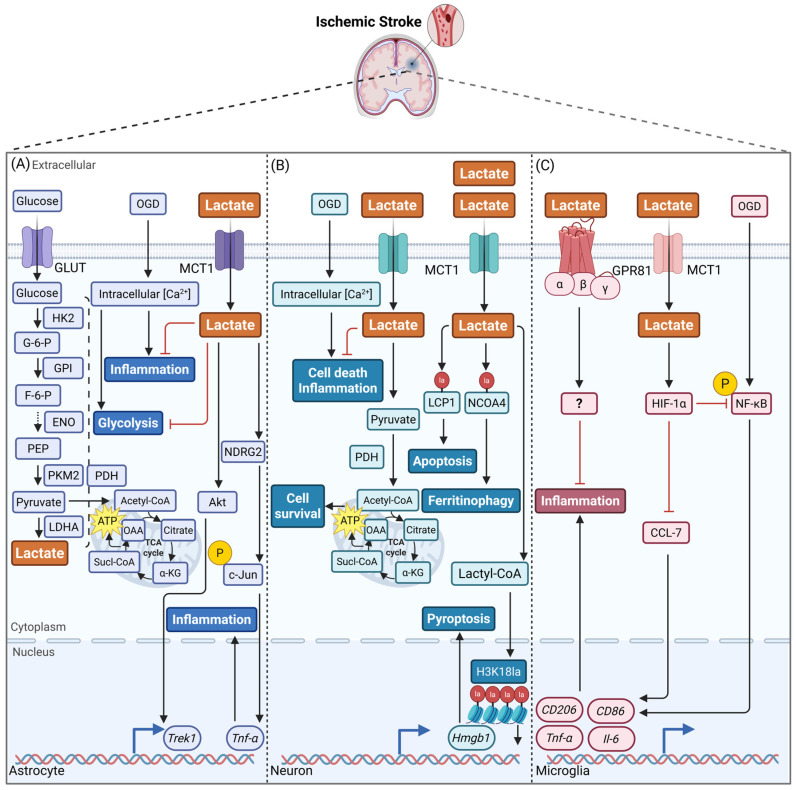
The regulatory role of lactate in the pathology of stroke and the mechanisms involved. (**A**) In astrocytes, OGD instigated a rapid escalation in intracellular Ca^2+^, ultimately resulting in cell death and the onset of pro-inflammatory alterations. Lactate has been demonstrated to provide astrocytes with protection against these deleterious effects, and this protection is accompanied by the inhibition of glycolysis and the activation of respiration [112]. In addition, lactate has been shown to inhibit the OGD-induced ubiquitination of NDRG2 in reactive astrocytes, thereby maintaining its stable expression and preventing its abnormal elevation [203]. Furthermore, lactate enhanced the expression of TREK1 in astrocytes during ischemia through the PKA pathway. These TREK1 channels have been demonstrated to contribute to neuroprotection by enhancing potassium buffering and promoting glutamate clearance in astrocytes [206]. (**B**) In neurons, lactate has been observed to regulate OGD-induced abnormalities in intracellular Ca^2+^ levels [112] and to facilitate the lactylation of histone proteins such as H3K18la, which may regulate pyroptosis by targeting HMGB1 [209]. Furthermore, lactylation of non-histone proteins, such as LCP1 and NCOA4, was found to be involved in the regulation of apoptosis and ferritinophagy [217,218]. (**C**) In microglia, activation of GPR81 reduced the inflammatory response during OGD, although the downstream signaling pathways remained unknown [111]. Lactate has also been observed to activate the CCL7/NF-κB signaling pathway via HIF-1α, leading to the downregulation of inflammatory genes during OGD/R [114,115]. This figure was created using BioRender (https://app.biorender.com/, accessed on 24 November 2025). Abbreviations: ↑, increase or activation; ⊥, decrease or inhibition; ⇢, a brief presentation of the facilitation mechanism; ?, the molecular signal has not been confirmed. 3,5-DHBA, 3,5-dihydroxybenzoic acid; ATP, adenosine triphosphate; CCL7, chemokine (C-C motif) ligand 7; CD, cluster of differentiation; c-Jun, Jun oncogene; GPR81, G protein-coupled receptor 81; IL-6, interleukin 6; LCP1, lymphocyte cytosolic protein 1; MCT, monocarboxylate transporters; NCOA4, nuclear receptor coactivator 4; NDRG2, N-myc downstream regulated gene 2; NF-κB, nuclear factor κB; OGD/R, oxygen-glucose deprivation/reoxygenation; PKA, protein kinase A; TNF-α, tumor necrosis factor α; TREK1, TWIK-related K^+^ channel 1.

It is important to note that there is an ongoing debate regarding the role of GPR81 in hypoxic-ischemic brain injury. A number of studies have suggested that GPR81 activation may underpin the neuroprotective effects of lactate in ischemic conditions by reducing lesion volume and promoting angiogenesis [197,198]. In contrast, alternative research suggests that the neuroprotective effects of lactate are not exclusively dependent on the GPR81 pathway. Rather, these effects may be influenced by regulation of energy metabolism or alternative signaling pathways [199,200]. In ex vivo oxygen-glucose deprivation (OGD) models, lactate supplementation has been shown to enhance neuronal tolerance to hypoxic injury by activating ANLS [201,202,203]. The following specific mechanisms have been identified: firstly, the inhibition of OGD-induced glutamate release and calcium influx to mitigate excitotoxicity [204]; secondly, the enhancement of astrocytic TWIK-related potassium channel 1 (TREK1) function via PKA signaling to improve potassium homeostasis and glutamate clearance [205] (Figure 4A); and thirdly, the direction of cellular metabolism towards aerobic oxidation while suppressing LPS-induced inflammatory responses by regulating intracellular calcium homeostasis and metabolic reprogramming [112] (Figure 4A,B). The N-myc downstream regulated gene 2 (NDRG2) has been identified as a member of the N-myc downstream regulated gene family, which is primarily involved in cell proliferation and differentiation. In astrocytes, NDRG2 has been demonstrated to exert a regulatory function in diverse processes, including apoptosis, astrogliosis, BBB integrity, and glutamate clearance [206]. In models of OGD and reoxygenation (OGD/R), increased expression of NDRG2 has been observed in astrocytes. Overexpression of NDRG2 has the potential to exacerbate the disruptions in glucose metabolism that are induced by OGD/R. These disruptions encompass diminished glucose uptake, augmented lactate production, and diminished levels of nicotinamide adenine dinucleotide phosphate (NADPH)/NADP^+^ and ATP. Furthermore, NDRG2 has been shown to induce alterations in iron metabolism within astrocytes by increasing ROS, iron, and COX2 levels, while decreasing glutathione (GSH) and glutathione peroxidase 4 (GPX4) [207]. In contrast, exogenous lactate treatment has been demonstrated to impede the OGD-induced ubiquitination of NDRG2 in reactive astrocytes, thereby preserving its stable expression and averting its aberrant upregulation [202] (Figure 4A). In microglia, the presence of lactate and 3,5-DHBA has been observed to counteract OGD-induced intracellular acidification and TNF-α release during reoxygenation [111]. Lactate treatment has been shown to reduce CCL7 expression through HIF-1α-mediated suppression of NF-κB signaling, thereby reducing neuroinflammation [114,115] (Figure 4C). As demonstrated by extant research, lactate exerts neuroprotective effects in cerebral ischemic injury through multiple mechanisms, including the provision of energy support, the inhibition of excitotoxicity, the regulation of ion homeostasis, and the modulation of inflammatory responses. Subsequent studies have sought to further elucidate the specific role of the GPR81 receptor in different cell types and the progression of ischemic pathology, whilst also exploring the molecular pathways through which lactate regulates the stability of key factors such as NDRG2.

Lactylation has been demonstrated to play a pivotal regulatory role in the pathological progression of IS. In NSCs, L-lactate modulates the Ski-related oncogene (SnoN) signaling axis via H3K9la, thereby promoting neurogenesis under hypoxic conditions [208]. In contrast, in neurons, hypoxic-reperfusion injury can result in the upregulation of LDH activity and the elevation of H3K18la levels. This, in turn, has been shown to induce pyroptosis through the activation of the high mobility group box-1 protein (HMGB1) signaling pathway [209]. Astrocyte-specific knockout of the *Ldha* gene has been demonstrated to reduce Pan-Kla levels and diminish cerebral infarct volume in a MCAO mouse model. Furthermore, the P300 inhibitor A-485 has been shown to mitigate neuronal death and glial activation, consequently extending the reperfusion treatment time window [210]. Lactylation in microglia has been observed to exhibit dynamic changes, wherein Suppressor of Mek1 (SMEK1), acting as a regulatory subunit of protein phosphatase 4, has been shown to promote lactate production upon its absence by inhibiting the PDK3-PDH pathway. This increase in H3K9la levels has been demonstrated to activate *Ldha* and *Hif-1α* transcription, thereby enhancing glycolysis. Conversely, SMEK1 overexpression has been demonstrated to enhance neurological recovery by modulating lactate-mediated metabolic reprogramming [211,212]. Furthermore, compensatory elevation of lactate in the periventricular region following cerebral ischemia enhances H3K18la modification. This modulates the expression of the Plexin B2 (Plxnb2), thereby promoting an anti-inflammatory microenvironment and exerting neuroprotective effects [213]. Proteomics studies indicate that post-cerebral ischemia-reperfusion, proteins such as voltage-gated anion channel 1 and SLC25A4/5 undergo lactylation modifications in ECs, contributing to calcium signaling dysregulation [214,215]. With regard to pharmacological interventions, buyang huanwu (BHD) has been shown to inhibit glycolysis and apoptosis by suppressing pan-luminal and H3K18la modifications in cerebral microvascular ECs, thereby antagonizing the transcriptional activity of apoptosis-activating factor 1 [216]. The regulatory role of non-histone lactylation in ischemic injury has garnered increasing attention. Research findings indicate that lactylation levels of lymphocyte cytosolic protein 1 (LCP1) exhibit a substantial increase following ischemia. In contrast, the glycolysis inhibitor 2-deoxy-D-glucose (2-DG) has been observed to reduce its lactylation modification, promote LCP1 degradation, and alleviate neuronal injury [217]. Lactylation of nuclear receptor coactivator 4 (NCOA4) at position K450 has been demonstrated to mediate hypoxia-induced glycolytic enhancement and ferritinophagy, thereby accelerating ischemic injury [218]. Intervention at this site has been shown to be an effective means of mitigating cerebral infarction. Furthermore, lactylation of Methyl-CpG-binding protein 2 (MeCP2) at K210/K249 has been shown to inhibit the transcription of pro-apoptotic genes, thereby reducing neuronal apoptosis [219]; whereas lactylation of ADP-ribosylation factor 1 (ARF1) at K73 has been demonstrated to participate in the regulation of mitochondrial release, influencing post-ischemic neuroprotective effects [220]. Collectively, these findings demonstrate that targeted modulation of non-histone lactylation, specifically LCP1, NCOA4, MeCP2, and ARF1, represents a promising strategy for mitigating IS pathology (Figure 4). Lactylation has been demonstrated to play a pivotal role in ischemic brain injury by regulating metabolic reprogramming, cell death, and neuroimmune responses. Elucidation of the integrative function of lactylation in intercellular communication within neurovascular units has the potential to provide a novel paradigm for precision interventions in stroke therapy.

In the context of hemorrhagic stroke, the results of studies utilizing animal models demonstrate significantly elevated lactate concentrations within the core and surrounding regions of cerebral hemorrhagic lesions. The efficacy of emodin intervention in reducing these levels has been demonstrated. Subsequent in vitro experiments have provided further evidence to support the initial finding that lactate promotes proliferation, migration, survival, and phagocytic function in microglia [221]. It is also indicated by additional research that lactate accumulation induces chemotaxis and aggregation of microglia by activating the NF-κB signaling pathway, thereby promoting angiogenesis and neurogenesis [125]. Furthermore, the inhibition of H3K14la has been shown to alleviate intracellular calcium overload and ferroptosis, thereby mitigating neuronal degeneration and improving neurological functional outcomes in mouse models of intracerebral hemorrhage (ICH) [222]. In the context of subarachnoid hemorrhage (SAH), elevated lactate levels in CSF have been shown to correlate with an early high glycolysis-lactate production pattern. This has been associated with favorable long-term recovery outcomes [223]. It has been determined that initial reductions in CBF, in conjunction with elevated lactate-to-pyruvate ratios, have the potential to serve as early warning indicators for the occurrence of delayed cerebral ischemia [224]. At the molecular level, bromodomain-containing protein 4 (BRD4), a key epigenetic regulator, has been shown to exert neuroprotective effects following SAH. Research indicates that astrocytes exhibit enhanced glycolysis and increased lactate production post-SAH to sustain cerebral energy supply. Silencing the BRD4 in astrocytes has been demonstrated to reduce lactate and H4K8la levels, thus promoting polarization towards a neurotoxic A1 phenotype and exacerbating neurological deficits [225]. It is evident that lactate fulfils a dual role in the metabolic reprogramming and neurorepair that occurs in the brain following a hemorrhagic stroke. The first function of lactate is to participate in the regulation of inflammation and cellular function. The second function is to serve as an energy substrate, thereby supporting the repair of brain tissue.

Physical exercise performs a multifaceted function in the promotion of stroke rehabilitation. A substantial body of clinical research has demonstrated that regular exercise exerts a proven effect in restoring muscle strength, endurance, coordination and balance in affected limbs [226,227]. Furthermore, it has been demonstrated that exercise promotes neural network remodeling in the brain, thereby strengthening neural connections and enhancing motor control and sensory function [228]. The biological mechanisms underlying these rehabilitative benefits have been shown to involve the release of neurotrophic factors, enhanced synaptic plasticity, angiogenesis, and reduced inflammatory and oxidative stress responses [229]. It has been demonstrated that exercise interventions can stimulate lactate production, thereby accelerating overall recovery. A systematic review has demonstrated that HIIT enhances plasma lactate, BDNF and VEGF levels, thereby participating in the regulation of neuroplasticity and promoting neuronal recovery following ischemic injury [230]. Moreover, acute HIIT has been shown to elevate serum BDNF levels and corticospinal excitability in chronic stroke patients [129,130]. This process is thought to be dependent on the regulatory effects of lactate on BDNF and cortisol levels. Furthermore, a four-week walking program significantly improved patients’ inhibitory control, with greater gains observed in individuals exhibiting higher lactate levels, suggesting that lactate may exert a regulatory role in exercise-induced enhancement of executive function [131]. In animal studies, a two-week period of moderate-to-high intensity treadmill training increased plasma lactate and corticosterone levels, accompanied by improved neurobehavioral scores. Furthermore, the training resulted in reduced infarct volume, enhanced neuronal density and synaptic plasticity, and cognitive recovery [132] (Table 6). However, other studies have indicated that long-term running training or exogenous lactate supplementation prior to stroke did not significantly improve cortical atrophy in post-stroke mice, and that this process was unrelated to GPR81 activation [231]. This finding indicates that the effects of lactate may be contingent on specific post-stroke pathophysiological contexts. The current body of research supports the notion that lactate plays a pivotal role in facilitating stroke recovery via exercise. The mechanisms through which it exerts its effects are manifold, including, but not limited to, neuroplasticity, metabolic regulation and inflammation control. It is recommended that subsequent research efforts concentrate on elucidating the dynamic mechanisms of lactate across the various stages of stroke rehabilitation. This should be achieved by means of a systematic comparison of the differential effects of varying exercise intensities and intervention timings on lactate signaling pathways and neurological outcomes, with due consideration for time-window specificity.

### 3.3. Lactate in Spinal Cord Injury and Exercise Intervention

**Figure 5:**
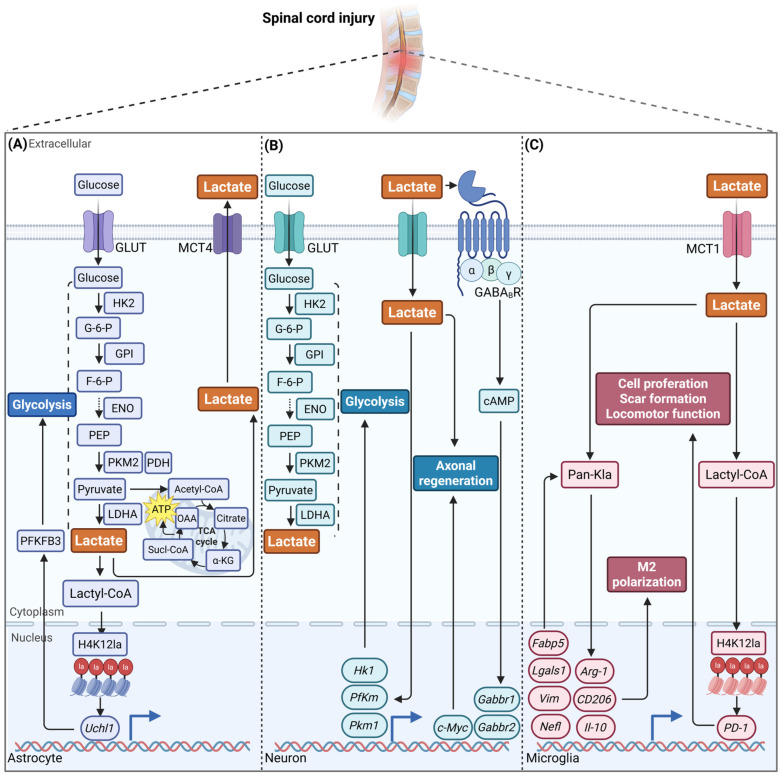
The regulatory role of lactate in the pathology of spinal cord injury and the mechanisms involved. (**A**) In astrocyte, the UCHL1/PFKFB3 axis has been demonstrated to enhance lactate production, thereby inducing histone lactylation and subsequent transcription of UCHL1 and several glycolysis-related genes. This observation suggests the existence of a glycolysis/H4K8la/UCHL1 positive feedback loop [240]. (**B**) In neurons, lactate has been observed to increase cAMP signaling through its action on GABA_B_R, thereby promoting axonal regeneration [14]. In addition, neurons utilize lactate to increase glycolysis released by ECs [234]. (**C**) In microglia, lactate has been shown to enhance the overall lactylation level and promote M2 polarization [134]. Furthermore, lactate-mediated H4K12la elevation has been shown to promote PD-1 transcription in microglia, thereby facilitating SCI repair [238]. This figure was created using BioRender (https://app.biorender.com/, accessed on 24 November 2025). Abbreviations: ↑, increase or activation; ⇢, a brief presentation of the facilitation mechanism; cAMP, cyclic adenosine monophosphate; GABA, γ-aminobutyric acid; MCT, monocarboxylate transporters; PD-1, programmed death 1.

The spinal cord constitutes a vital component of the CNS. It serves as the primary conduit for neural signal transmission and regulates reflexes and fundamental motor functions. In animal models of SCI, elevated lactate levels and increased lactate-to-pyruvate ratios have been observed in both CSF and spinal cord tissue [232,233]. In addition, human studies have demonstrated a positive correlation between motor function scores, spinal cord perfusion pressure and the lactate-to-pyruvate ratio [18], suggesting that lactate metabolism plays a crucial role in the functional recovery of the spinal cord. The administration of exogenous lactate has been shown to activate GABA_B_R, thus promoting axonal regeneration through the modulation of cAMP signaling and the enhancement of motor function in murine models [14] (Figure 5A). Concurrently, the restoration of the expression of downregulated endothelial MCT1 post-injury facilitates lactate transport to neurons, thereby enhancing the capacity for axonal regeneration [234]. In the aftermath of SCI, the elevated levels of lactate observed in astrocytes are attributable to abnormally activated ANLS within the context of chronic pain mechanisms. This phenomenon has been demonstrated to contribute to the sustenance of mechanical pain thresholds [235]. Exogenous lactate has been demonstrated to induce spinal LTP and hyperalgesia [236]. Impaired glycolysis in microglia following injury can be counteracted by lactate supplementation and lactylation, promoting their polarization towards an anti-inflammatory M2 phenotype [134]. Further studies have indicated that lactylation in microglia and macrophages is a driving factor in the transcription of the chemokine CXCR16. This, in turn, has been shown to recruit CD8^+^ T cells, which have been found to exacerbate neuronal loss. The targeting of this pathway has been demonstrated to facilitate the recovery of motor function [237]. At the epigenetic level, lactate has been observed to increase H4K12la levels in microglia, thereby upregulating programmed cell death 1 (PD-1) expression and promoting cell proliferation, scar formation and axonal regeneration [238]. Furthermore, H4K12la has been demonstrated to enhance secreted phosphoprotein 1 (SPP1) transcription, thereby promoting axonal mitochondrial function and recovery [239]. Moreover, deubiquitinase ubiquitin C-terminal hydrolase-L1 (UCHL1) in astrocytes has been demonstrated to promote lactate production by stabilizing PFKFB3, which in turn triggers H4K8la modification. This forms a positive feedback loop that facilitates metabolic reprogramming and protects neuronal survival [240]. These findings provide preliminary insights into the role of lactate in SCI repair and its underlying mechanisms. However, further exploration is required to achieve a comprehensive understanding of the mechanisms by which lactate promotes SCI repair through lactylation and regulates microglial function. In addition, there is a necessity for further research in the form of larger-scale preclinical and clinical studies is required in order to validate the therapeutic potential of lactate in the recovery of various types of SCI.

Evidence suggests that SCI frequently results in loss of limb function, sensory deficits, and problems with excretory function, among other consequences [241]. The mechanisms by which exercise exerts its effects are numerous. Specifically, it has been demonstrated that exercise increases neuroplasticity, stimulates functional remodeling of the spinal cord and cerebral cortex, improves lost motor control and sensory function, and promotes nerve regeneration and repair at the site of injury [242]. In addition, the capacity of exercise to modulate the inflammatory response, and attenuate neuropathic pain following SCI is further evidence of its potential to contribute to neuroprotection and repair [243]. Early research in the field of human studies has demonstrated that blood lactate levels are significantly elevated in patients with SCI following exercise [133], and that the magnitude of elevated lactate levels is strongly correlated with exercise load [244,245]. However, there is an absence of immediate clinical evidence confirming a link between exercise-induced elevations in circulating lactate levels and functional recovery in patients with SCI. A recent study utilizing an animal model demonstrated that a three-day treadmill running program prior to SCI modelling resulted in a significant increase in lactate levels in the serum and spinal cord of mice. Furthermore, this exercise program has been shown to significantly increase Pan-Kla levels in microglia, while concomitantly downregulating the mRNA expression of SCI-induced inflammatory factors, including IL-1β, iNOS, and TNF-α. In addition, the exercise program has been demonstrated to elevate motor scores and promote functional recovery [134] (Table 6). Further validation is required through subsequent studies to substantiate the association between exercise, lactate, and functional recovery in SCI in human experiments. The investigation of how exercise-induced alterations in lactate metabolism can facilitate functional recovery by modulating inflammatory responses and nerve repair should be a central focus.

## 4. Limitations and Future Perspectives

### 4.1. Examining the “Dual-Edge” Effects of Lactate

This review comprehensively elucidates the multifaceted functions of lactate within the CNS, with particular emphasis on its potential pivotal role in exercise-mediated neuroprotection. It is imperative to acknowledge the context-dependency of the biological effects of lactate, whereby its direction of action is determined by local concentration, temporal window of action, and the specific pathological microenvironment. This phenomenon is especially evident in the context of neuroinflammation regulation. A substantial body of research suggests that, under specific conditions, lactate may exhibit anti-inflammatory and neuroprotective properties. For instance, it has been demonstrated that this process may induce synaptic elongation in microglia by activating the Akt signaling pathway, thereby suppressing LPS-triggered pro-inflammatory responses and alleviating depression-like behavior [99]. In models of cerebral ischemia, exogenous lactate supplementation has been shown to upregulate HIF-1α expression within microglia, inhibit the CCL7/NF-κB signaling pathway, and promote microglial polarization towards the anti-inflammatory M2 phenotype. This, in turn, has been demonstrated to mitigate neuroinflammation and brain injury following ischemia-reperfusion [114]. Furthermore, post-ischemic elevation of H3K18la has been demonstrated to promote the formation of an anti-inflammatory microenvironment by regulating the expression of *Plxnb2* in microglia [213]. In astrocytes, the presence of lactate under conditions of hypoxia and hypoglycemia has been shown to stabilize NDRG2 protein, thereby inhibiting c-Jun-dependent phosphorylation of TNF-α expression and secretion, thus exerting anti-inflammatory effects [203].

However, in other pathological contexts, lactate has been observed to exacerbate neuroinflammation. The extant research suggests that L-lactate has the capacity to reduce microglial phagocytic capacity via the GPR81 pathway. Furthermore, it has been demonstrated that this compound exerts a significant synergistic effect with LPS, resulting in the substantial upregulation of pro-inflammatory genes. This finding indicates a possible role in the promotion of microglial polarization towards the pro-inflammatory M1 phenotype [109]. It is important to note that the effects of lactate may exhibit opposing roles across different disease phases. For instance, during the ischemic phase of stroke, astrocyte-derived lactate exacerbates brain injury by promoting neuronal Kla. In contrast, the administration of lactate during reperfusion has been demonstrated to exhibit significant neuroprotective effects, underscoring its time-window-dependent functionality [114,246]. Furthermore, the function of the GPR81 in acute injury models, such as cerebral ischemia, demonstrates considerable divergence, thereby further elucidating the intricacy of its regulatory mechanisms. It has been hypothesized that the initial activation of GPR81 during periods of ischemia may promote neurogenesis and cerebral angiogenesis, thus exerting a protective effect [198]. However, evidence also indicates that GPR81 activation may trigger conflicting responses in different cell types, potentially exacerbating injury [25]. This inconsistency may be attributable to variations in experimental models (e.g., permanent versus transient ischemia), intervention timing (acute versus recovery phase), and cell specificity. Consequently, future research must move beyond the simplistic binary ‘beneficial or harmful’ narrative and strive to construct a dynamic framework capable of predicting and precisely regulating lactate’s functional orientation. This necessitates the development of tools with high spatiotemporal resolution, such as chemogenetic sensors capable of real-time monitoring of lactate dynamics in specific brain regions. The utilization of such instruments would enable the precise mapping of quantitative relationships between lactate concentration gradients and cellular responses during distinct phases of cerebral ischemia (acute ischemia and reperfusion) or specific progression stages of chronic neurodegenerative diseases. This would ultimately define the critical thresholds at which lactate transitions from neuroprotection to neurotoxicity.

### 4.2. Deepening Research into the Mechanisms and Functions of Lactylation Modification

Exercise-induced lactylation has been demonstrated to exert key regulatory roles in diverse physiological processes by targeting specific histones and non-histones. Among non-histone modifications, it is notable that lactylation of YTH domain family member 2 modulates cardiac adaptation and protection via Ras GTPase-activating protein-binding protein 1 in cardiomyocytes [247]. It has been demonstrated that MeCP2 K271 lactylation exerts a beneficial effect on atherosclerosis by inhibiting the epiregulin-MAPK pathway [248]. In addition, mTOR K921 lactylation has been shown to promote skeletal muscle autophagy by inactivating mTORC1 [249]. Concurrently, the synaptosome-associated protein 91 lactylation has been found to enhance synaptic function in order to counteract anxiety-like behavior [39]. In relation to histone modifications, H3K18la has been demonstrated to promote bone formation by upregulating osteogenic genes, such as collagen type I alpha 2 chain and cartilage oligomeric matrix protein [40]. In contrast, the maintenance of Pan-Kla levels has been demonstrated to counteract cellular and muscular ageing by activating DNA repair and protein homeostasis pathways [250]. Furthermore, lactylation has been observed to promote a transition in glial cells towards an anti-inflammatory/repair phenotype, thus enhancing neurological function [47]. Collectively, these findings reveal lactylation as a pivotal adaptive signal in protective regulation across cardiovascular, skeletal, muscular, and neurological systems. However, the dynamic characteristics of lactylation in response to physical exercise, including its sites of modification, abundance, timing, and duration, remain highly intricate and necessitate further extensive research. Furthermore, extant research endeavors have encountered difficulties in establishing exercise prescriptions that are in alignment with lactylation response patterns. To date, there has been a paucity of systematic exploration with regard to the differential regulation of the Pan-Kla landscape by different exercise interventions (e.g., intensity, duration and type).

In order to surmount the present limitations in research, it is imperative to establish a systematic evidence framework that is multi-layered and integrates multi-omics data with cutting-edge methodologies. In particular, single-cell RNA sequencing should be combined with spatial transcriptomics in order to precisely identify altered gene expression profiles within specific brain regions during exercise-induced lactate dynamics [251]. This approach will target neuronal, astrocytic, and microglial subpopulations. Moreover, transcriptomic data should be coupled with mass spectrometry-based lactylation omics results. Correlating the dynamics of lactylation modification with gene transcriptional regulation patterns facilitates the discernment of dominant biological events from accompanying phenomena [252,253]. This, in turn, enables the identification of lactylation targets that are critical to exercise-mediated neuroprotection. Building upon this, multi-factor orthogonal experimental designs should be introduced to systematically manipulate exercise intensity, duration, and type within disease models. The integration of phenotypic indicators, such as cognitive improvement and neuroinflammation alleviation, facilitates the elucidation of the characteristics of tissue-specific lactylation modifications and transcriptional responses. This approach will establish dose-response relationships between exercise parameters, specific lactylation sites, and neurological functional improvements, providing a theoretical foundation for precision exercise interventions. In the context of mechanism validation, there is a compelling imperative to proactively advance and implement CRISPR-dCas9-based epigenomic editing tools and cell-specific knockout animal models. It is anticipated that this approach will enable precise regulation of specific lactylation modifications and key enzyme activities in vivo, thereby elucidating their functional contributions. It is imperative that efforts are concentrated on the development of AI-driven deep proteomics platforms to systematically uncover novel post-translational modifications [254,255,256,257]. The employment of chemical proteomics methodologies promises a more profound and nuanced understanding of the regulatory mechanisms and functions of lactylation in pathological contexts, such as cancer and neurodegenerative diseases. The possession of such knowledge has the potential to facilitate the development of targeted intervention strategies. Concurrently, there is a need for AI-driven clinical proteomics research to advance to a stage of in-depth exploration of disease biomarkers and drug targets. This will provide critical technological support for the eventual realization of precision medicine based on lactylation regulation.

### 4.3. Advancing the Translation of Lactate Research from Models to Clinical Practice

It is also noteworthy that current mechanisms discovered and understood in this field primarily stem from research using animal models. Undeniably, animal model studies serve as a foundational platform for understanding the complex signaling and functional roles of lactate in the central nervous system. The utilization of transgenic, knockout, and disease-specific models (e.g., AD, IS, and SCI) in research endeavors furnishes invaluable causal evidence that is otherwise challenging to procure directly from human subjects. This evidence is crucial for elucidating the specific cellular and molecular mechanisms by which lactate regulates the central nervous system. These models facilitate precise manipulation of specific genes, receptors, transporters and lactylation pathways, enabling direct assessment of their impact on neuroprotection and functional recovery. Consequently, notwithstanding the evident species differences, the evidence from the animal model highlighted in this review demonstrates the multifaceted role currently attributed to lactate within the CNS as an energy substrate, signaling molecule, and epigenetic regulator. Conversely, the utilization of animal studies is encumbered by a plethora of limitations. Firstly, it is important to note that rodents exhibit marked differences from humans in terms of the complexity of their nervous systems and metabolic pathways. Such species-specific variations may result in the manifestation of distinct sensitivities, specificities, and network regulatory mechanisms in response to lactate. Secondly, animal models are typically employed to simulate specific aspects or acute phases of disease. It is evident that simplified disease models are incapable of fully replicating the prolonged, chronic and heterogeneous pathophysiological processes that are characteristic of human central nervous system diseases, such as AD. In conclusion, while the utilization of animal models in research facilitates the standardization of exercise protocols and lactate administration methods to a considerable extent, human research is distinguished by a greater degree of complexity in the evaluation of intervention effects. This phenomenon can be attributed to a number of factors, including individual variability, compliance with treatment guidelines, and the presence of comorbidities.

Subsequent research, having established preliminary evidence in rodent models, must systematically evaluate translational potential in larger animal models more closely resembling human physiology, such as non-human primates. Concurrently, human-derived in vitro models, such as neurons and glial cells derived from human induced pluripotent stem cells, should be utilized to validate the core mechanisms of lactate-related signaling pathways and epigenetic modifications within environments simulating human genetic backgrounds, pathological features and cellular interactions. Furthermore, the advancement of prospective clinical translational studies is of the utmost importance. Despite the considerable research that has been dedicated to investigating the efficacy of exercise interventions in addressing blood lactate and CNS diseases, the current evidence base remains inadequate [175,227,258]. It is imperative that rigorous, large-scale, randomized controlled trials are conducted with a high degree of urgency in order to ascertain their efficacy. In this regard, large-scale, meticulously designed prospective clinical cohort studies should be conducted, with a focus on the feasibility of peripheral blood lactate kinetic parameters (such as peak concentration and clearance rate) as potential biomarkers for predicting the efficacy of exercise interventions in specific CNS diseases. It is imperative to acknowledge the advancement of mechanism-based precision clinical trials, which holds greater significance in the context of medical research and development. The identification of suitable patient subgroups on the basis of particular targets disclosed by basic research (e.g., GPR81 activation or key protein lactate modification) would be a prerequisite for the implementation of these methods. The implementation of standardized exercise protocols or exploratory drug interventions would serve to directly validate the causal role of the ‘lactate signaling axis’ in disease treatment within human subjects. For patients unable to undergo effective exercise interventions due to functional impairments, the development of alternative strategies that mimic the benefits of exercise is a crucial direction in translational medicine. However, the inherently brief half-life and rapid metabolism of lactate give rise to significant pharmacokinetic challenges in terms of its direct use as a therapeutic agent [259,260,261,262]. In order to overcome this obstacle, future research may focus on two delivery strategies: firstly, developing targeted delivery systems based on nanocarriers. These could potentially encapsulate lactate or its prodrug precursors, with surface functionalization enhancing BBB penetration and tissue-specific accumulation. The primary benefit of this approach would be an increase in the duration of action, with a concomitant improvement in bioavailability and a reduction in systemic off-target risks. A secondary advantage would be the exploration of nutritional or metabolic intervention approaches, which could prove to be a valuable addition to the existing body of knowledge in this field. The process involves the supplementation of specific precursor substances or the modulation of relevant enzyme activity. The objective is to regulate endogenous lactate levels and associated modification processes safely and controllably. It is imperative that rigorous evaluation of the specificity, long-term safety, and potential off-target effects of all intervention methods is undertaken throughout this process, in order to ensure the scientific validity and robustness of translational applications.

## 5. Conclusions

This review establishes the regulatory role of lactate-mediated exercise on the CNS, thereby effecting a profound shift in understanding of its biological function. Exercise-induced lactate production provides the brain with an efficient energy substrate and also exerts neuroprotective effects through multiple molecular mechanisms. These mechanisms encompass the following: the generation of lactate and its intercellular transport, which are mediated by glycolytic enzymes and MCTs; the activation of the GPR81; the induction of lactate-mediated processes; and the precise regulation of key signaling pathways, including ERK1/2, SIRT1/PGC-1α/FNDC5, and Akt/eNOS/VEGF pathways. It has been demonstrated that, under physiological conditions, lactate has a number of important functions in the CNS. These include the promotion of neurogenesis, synaptic plasticity, mitochondrial function, cerebral angiogenesis and the alleviation of neuroinflammation (Figure 2 and Table 1, Table 2, Table 3, Table 4 and Table 5). Furthermore, it has been demonstrated that lactate has the capacity to modulate the progression of various CNS diseases, including AD, IS and SCI (Figure 3, Figure 4 and Figure 5). Strategies such as exercise intervention that enhance lactate production may alleviate clinical symptoms of these conditions and play a pivotal role in early intervention and treatment (Table 6). In the future, it is only through the deep integration of neuroscience, exercise physiology, bioengineering and clinical medicine that lactate research can be advanced from the laboratory to clinical practice, thereby truly transforming lactate into an innovative force for enhancing human CNS health.

## Figures and Tables

**Table 1:** The effects and mechanisms of lactate on neurogenesis within the central nervous system.

Subjects and Samples	Treatments	Mechanism	Major Effects of Lactate	Reference
Mouse brain	L-lactate administration	MCT2-dependent manner	Number of NeuN^+^ BrdU^+^ cells in DG ↑	[58]
Mouse brain	*Mct2* and *Mct4* gene manipulation; L-lactate administration	MCT2-dependent manner	DCX expression ↑	[57]
Mouse brain; primary NPCs	*Pkm2*, *Gpr81*, *Mct2* gene manipulation; L-lactate administration	MCT2-dependent manner	Number of EdU^+^ cells, RGL NSPCs, transiently amplifying progenitor cells, BrdU^+^ DCX^+^ and BrdU^+^ NeuN^+^ cells in DG ↑	[10]
Mouse brain; primary leptomeningeal fibroblast	*Gpr81* gene manipulation; L-lactate administration; HIIT	Activation of GPR81 and downstream Akt pathway	Number of DCX^+^ cells, Ki-67^+^ cells, and DCX^+^ Ki-67^+^ cells in the V-SVZ ↑	[43]
Mouse brain; primary NPCs, neurons, and astrocytes; Human 293T and mouse N2a cell lines	*Mct1*, *Mct2* gene manipulation; voluntary running	Lactate-induced H4K12la and activation of MDM2-p53 pathway	Percent of BrdU^+^ cells and activated caspase-3^+^ cells ↑	[9]
Primary NPCs	L-lactate administration	Activation of ERK1/2 and Akt signaling	Percent of BrdU^+^ cells in DG ↑; glucose metabolism ↑	[56]
Neonatal mice	Exposures to sevoflurane; L-lactate administration	Activation of SIRT1/PGC-1α/FNDC5 pathway	Number of BrdU^+^ cell and BrdU^+^ DCX^+^ cells in SGZ ↑	[4]

Abbreviations: ↑, increase or activation; Akt, protein kinase B; BrdU, bromodeoxyuridine; DCX, doublecortin; DG, dentate gyrus; EdU, 5-ethynyl-2′-deoxyuridine; ERK, extracellular signal-regulated kinases; FNDC5, fibronectin type III domain-containing protein 5; GPR81, G-protein coupled receptor 81; HIIT, high-intensity interval training; H4K12la, histone H4 lysine 12 lactylation; Ki-67, antigen Ki-67; MDM2, mouse double minute 2 homolog; MCT, monocarboxylate transporter; NeuN, neuronal nuclei; NPC, neural progenitor cell; NSPC, neural stem/progenitor cell; PKB, also known as Akt, protein kinase B; PKM2, pyruvate kinase M2; PGC-1α, peroxisome proliferator-activated receptor γ coactivator 1α; RGL, radial glia-like cells; SGZ, subgranular zone; SIRT1, silent mating type information regulation 2 homolog-1; V-SVZ, ventricular-subventricular zone.

**Table 6:** The role of physical exercise in regulating lactate and its associated signals in patients or animal models of central nervous system diseases.

Subjects			Exercise Program			Major Results	Reference
Models	Sex	Age	Mode	Intensity	Duration		
AD patients	M/F	76.7 ± 7.0 years	Power cycling	45~55% HRR; 65~75% HRR	25 min	Blood lactate ↑; brain glucose metabolism ↓	[126]
C57/BL6J mice	M	10 weeks old	Treadmill running	20~40 m/s exercise to exhaustion	N/A	Blood lactate ↑; ADAM10 activity in the prefrontal cortex and hippocampal ↑; BACE1 activity in the prefrontal cortex ↓	[127]
APP/PS1 mice	M	3 months old	Treadmill running	7~15 m/s	45 min/session, 5 sessions/week for 12 weeks	GPR81, cAMP and PKA expression ↑; dendritic spine density, PSD-95, GluA1 and CaMKII expression ↑; microglia activation ↓; C1q and C3 generation ↓; cognition ↑	[128]
AlCl3/D-gal induced mice	M	13~14 weeks old	Treadmill running	70%_max_ running speed	40 min/session, 5 sessions/week for 8 weeks	Histone H3 lactylation ↑; microglia anti-inflammatory phenotype ↑; cognition ↑	[47]
Stroke patients	M/F	54.7 ± 9.7 years	Treadmill running; seated stepper	HIIT or MICT	20 min	Blood lactate ↑	[129,130]
Stroke patients	M/F	61 ± 11 years	Walking exercise	HIIT or MICT	40 min/session, 3 sessions/week for 4 weeks	Blood lactate ↑; inhibitory control ↑	[131]
MCAO rats	M	3 weeks old	Treadmill running	Low, medium or high intensity	30 min/session, 1 session/day for 2 weeks	Blood lactate and corticosterone ↑; neurobehavioral score and cerebral infarct volume ↓; neuronal density ↑; SYN and PSD-95 expression ↑; cognition ↑	[132]
SCI patients	M	30.3 ± 2.9 years; 27.8 ± 4.2 years	Friction-braked arm ergometer	Exercise to exhaustion	N/A	Blood lactate ↑	[133]
SCI mice	M	6~8 weeks old	Treadmill running	N/A	3 days	Serum and spinal cord lactate ↑; microglia Pan-Kla ↑; IL-1β, iNOS and TNF-α expression ↑; neuronal density ↑	[134]

Abbreviations: ↑, increase or activation; ↓, decrease or inhibition; 1RM, 1 repetition maximum; AD, Alzheimer’s disease; ADAM10, a disintegrin and metalloprotease 10; BACE1, β-site APP cleaving enzyme 1; BDNF, brain-derived neurotrophic factor; CaMKII, calcium/calmodulin-dependent protein kinase II; cAMP, cyclic adenosine monophosphate; GluA1, AMPA-selective glutamate receptor 1; GPR81, G protein-coupled receptor 81; HIIT, high-intensity interval training; HRR, heart rate reserve; IL-1β, interleukin 1β; iNOS, inducible nitric oxide synthase; MICT, moderate intensity continuous training; MCAO, middle cerebral artery occlusion; N/A, not available; PKA, protein kinase A; PSD-95, postsynaptic density protein 95; SCI, spinal cord injury; SYN, synaptophysin; TNF-α, tumor necrosis factor α.

## Data Availability

No data was used for the research described in the article.

## Abbreviations

The following abbreviations are used in this manuscript:

1RM	1 repetition maximum
2-DG	2-deoxy-d-glucose
3,5-DHBA	3,5-dihydroxybenzoic acid
3-OBA	3-hydroxybutyrate acid
Aβ	amyloid β-protein
AC	adenylate cyclase
ACC	anterior cingulate cortex
Acetyl-CoA	acetoacetyl coenzyme A
AD	Alzheimer’s disease
ADAM10	a disintegrin and metalloprotease 10
ADP	adenosine diphosphate
AHN	adult hippocampal neurogenesis
AGRP	agouti-related protein
Akt	protein kinase B
AlCl3	aluminum chloride
AMPK	adenosine 5′-monophosphate (AMP)-activated protein kinase
Ang	angiopoietin
ANLS	astrocyte-neuron lactate shuttle
APP	amyloid precursor protein
Arc	activity-regulated cytoskeletal
ARF1	ADP-ribosylation factor 1
ASCT2	alanine-serine-cysteine transporter 2
ATP	adenosine triphosphate
BACE1	β-site APP cleaving enzyme 1
BBB	blood-brain barrier
BDNF	brain-derived neurotrophic factor
BHD	buyang huanwu
BRD	bromodomain-containing protein
cAMP	cyclic adenosine monophosphate
CaMKII	calcium/calmodulin -dependent protein kinase II
CBF	cerebral blood flow
CCL	chemokine (C-C motif) ligand
CNS	central nervous system
CSF	cerebrospinal fluid
CX3CR1	chemokine (C-X3-C motif) receptor
DG	dentate gyrus
D-gal	D-galactosamine
DRP1	dynamin-related protein 1
EAAT	excitatory amino acid transporter
EC	endothelial cell
EGR1	early growth response protein 1
EndMT	endothelial-to-mesenchymal transition
eNOS	endothelial nitric oxide synthase
ERK	extracellular regulated protein kinases
FGF	fibroblast growth factor
FIS1	fission 1
FoxO3	forkhead box protein O3
FNDC5	fibronectin type III domain-containing protein 5
GABA_B_R	γ-aminobutyric acid B-type receptors
GAP	growth-associated protein
GLP-1	glucagon-like peptide-1
GLS	glutaminase
GluA1	AMPA-selective glutamate receptor 1
GLUT	glucose transporter
GPR81	G protein-coupled receptor 81
GPX4	glutathione peroxidase 4
GSH	glutathione
GSK-3β	glycogen synthase kinase 3β
GTPγS	guanosine 5′-O-(3-thiotriphosphate)
H3K18la	histone H3 lysine 18 lactylation
HCAR	hydroxy-carboxylic acid receptors
HDAC	histone deacetylase
HIF-1α	hypoxia-inducible factor 1α
HIIT	high-intensity interval training
HK	hexokinase
HMGB1	high mobility group box-1 protein
HRR	heart rate reserve
ICH	intracerebral hemorrhage
IDH3β	pyruvate dehydrogenase 3β
IDO1	indoleamine-2,3-dioxygenase 1
IGFBP	insulin-like growth factor binding protein
IL	interleukin
IS	ischemic stroke
JAK	Janus kinase
KAT	lysine acetyltransferase
KATP	ATP-sensitive potassium channel
KIF5B	kinesin family member 5B
KYN	kynurenine
LCP1	lymphocyte cytosolic protein 1
LDH	lactate dehydrogenase
LPS	lipopolysaccharide
LRRC15	leucine-rich repeat-containing 15
LTP	long-term potentiation
MAPK	mitogen-activated protein kinase
MCAO	middle cerebral artery occlusion
MCT	monocarboxylate transporter
MDD	major depressive disorder
MDM2	mouse double minute 2 homolog
MeCP2	methyl-CpG-binding protein 2
MICT	moderate-intensity continuous training
mTOR	mammalian target of rapamycin
NADH	nicotinamide adenine dinucleotide
NCOA4	nuclear receptor coactivator 4
NDRG2	N-myc downstream regulated gene 2
NEK7	NIMA-associated kinase 7
NF-κB	nuclear factor κB
NFTs	neurofibrillary tangles
NLRP3	NLR family Pyrin domain protein 3
NMDAR	N-methyl-D-aspartate receptor
NO	nitric oxide
NPC	neural progenitor cells
NRF2	nuclear factor erythroid 2-related factor 2
NSC	neural stem cell
OGD	oxygen-glucose deprivation
OL	oligodendrocytes
OXPHOS	oxidative phosphorylation
PAH	pulmonary hypertension
PAX	pannexin
PD-1	programmed cell death 1
PDGF	platelet-derived growth factor
PDH	pyruvate dehydrogenase
PGC-1α	peroxisome proliferators-activated receptor γ coactivator lα
PFK	phosphofructokinase
PFKFB3	6-phosphofructo-2-kinase/fructose-2,6-bisphosphatase 3
PI3K	phosphatidylinositol 3-kinase
PINK1	PTEN-induced kinase
PKA	protein kinase A
PKM2	pyruvate kinase isozymes M2
PLC-γ	phospholipase C-γ
Plxnb2	plexin B2
POMC	proopiomelanocortin
PSD-95	postsynaptic density 95
PTEN	phosphatase and tensin homolog
PPAR	peroxisome proliferator-activated receptor
PTM	post-translational modifications
RE	resistance exercise
ROS	reactive oxygen species
SASP	senescence-associated secretory phenotype
SAH	subarachnoid hemorrhage
SCI	spinal cord injury
SGZ	subgranular zone
SIRT	silent mating type information regulation 2 homolog
SMEK1	suppressor of Mek1
STAT	signal transducer and activator of transcription
SVZ	sub-ventricular zone
SYN	synaptophysin
T2DM	type 2 diabetes mellitus
TCA	tricarboxylic acid
TNF-α	tumor necrosis factor α
TREK	TWIK-related K^+^ channel
TrκB	tropomyosin receptor kinase B
TRP	tryptophan
TSP-1	thrombospondin 1
VEC	vascular endothelial cell
VEGF	vascular endothelial growth factor
VGLL4	vestigial-like family member 4
VSMC	vascular smooth muscle cell
V-SVZ	ventricular-subventricular zone
YY1K183la	Yin Yang-1 lysine 183 lactylation
